# Estimating neuronal connectivity from axonal and dendritic density fields

**DOI:** 10.3389/fncom.2013.00160

**Published:** 2013-11-25

**Authors:** Jaap van Pelt, Arjen van Ooyen

**Affiliations:** Computational Neuroscience Group, Department of Integrative Neurophysiology, Center for Neurogenomics and Cognitive Research, VU University AmsterdamAmsterdam, Netherlands

**Keywords:** neuronal morphology, density fields, synaptic connectivity, 3D line crossing, random lines, intersections of cubes

## Abstract

Neurons innervate space by extending axonal and dendritic arborizations. When axons and dendrites come in close proximity of each other, synapses between neurons can be formed. Neurons vary greatly in their morphologies and synaptic connections with other neurons. The size and shape of the arborizations determine the way neurons innervate space. A neuron may therefore be characterized by the spatial distribution of its axonal and dendritic “mass.” A population mean “mass” density field of a particular neuron type can be obtained by averaging over the individual variations in neuron geometries. Connectivity in terms of candidate synaptic contacts between neurons can be determined directly on the basis of their arborizations but also indirectly on the basis of their density fields. To decide when a candidate synapse can be formed, we previously developed a criterion defining that axonal and dendritic line pieces should cross in 3D and have an orthogonal distance less than a threshold value. In this paper, we developed new methodology for applying this criterion to density fields. We show that estimates of the number of contacts between neuron pairs calculated from their density fields are fully consistent with the number of contacts calculated from the actual arborizations. However, the estimation of the connection probability and the expected number of contacts per connection cannot be calculated directly from density fields, because density fields do not carry anymore the correlative structure in the spatial distribution of synaptic contacts. Alternatively, these two connectivity measures can be estimated from the expected number of contacts by using empirical mapping functions. The neurons used for the validation studies were generated by our neuron simulator NETMORPH. An example is given of the estimation of average connectivity and Euclidean pre- and postsynaptic distance distributions in a network of neurons represented by their population mean density fields.

## Introduction

Because synapses can form only when axons and dendrites are in close proximity, the connectivity in neuronal networks strongly depends on the three-dimensional morphology of the constituting neurons. Neuronal morphology varies greatly, and the substantial variability in neuronal morphologies will consequently also produce large variability in their connections with other neurons. An additional factor determining connectivity is the spatial position of neurons, leading to widely varying distances between neurons pairs. The morphology of neurons is complex, with branches of varying orientations and diameters bifurcating at different lengths. In reconstructions this complex morphology is usually approximated in a piece-wise linear fashion, i.e., by a number of line pieces or cylinders (the latter when the diameter is also measured). These reconstructions in continuous space preserve the details of the arbor structures of the neurons. Another way of characterizing the spatial structure of neurons is by discretizing space by means of a grid of voxels and defining in each voxel the neuronal “mass” (i.e., the length or the volume of a branch in that voxel). When the mass in each voxel is divided by the voxel volume, this description results in a neuronal “mass” density field (in short called density field). Clearly, the density field of a single neuron fully reflects the arbor structure of the neuron, with non-zero densities in voxels occupied by arbors and zero densities elsewhere.

When an average density field is obtained from a number of neurons (after alignment of the somata), the individual arbor structures get lost, and the number of non-zero voxel densities increases because of the large variations in neuronal morphologies. Only for very high neuron numbers will a stable estimate of the population mean density field be obtained. Although the level of smoothness of the population mean density field may be high in areas near the soma, it will remain low in remote areas, which are visited only by spurious branches of individual neurons. The smoothness of a density field may be enhanced if certain symmetries can be assumed in the averaged morphology of cells. For instance, when neurons grow out without any orientation preference, a spherical symmetry in the density field may be assumed. In that case, the total mass at a certain radial distance from the soma can be smeared out uniformly over the sphere with that radius. Similarly, when rotation invariance around a central axis can be assumed, the total mass at a certain radial distance from, and a certain height at the axis, can be smeared out uniformly over the circle with that radius and at that height. Stable estimates of the population mean density fields of neurons reflect shape characteristics that are typical for a given cell type. Therefore, these estimates can be regarded as powerful statistical descriptors of the neurons' spatial innervation patterns and, as such, as templates for various neuronal cell types.

Synaptic contacts may occur when axonal and dendritic elements are very close in space, i.e., within a few microns, a condition usually referred to as Peters' rule (Peters, 1979). Binzegger et al. (2004) use another interpretation of Peters' rule in that axons connect in direct proportion to the occurrence of the synaptic target structures in the neuropil. Locations where candidate synapses can be formed can be found by testing the proximity of any pair of line pieces of the axonal and dendritic arborizations of neuronal reconstructions. Recently, we developed a new method for finding candidate synaptic locations in areas innervated by both axonal and dendritic arborizations. The method defines the precise locations of the candidate synaptic contact points on the axonal and dendritic segments. The term candidate is used because it refers to the minimal geometric requirement for a synapse. Whether in neuronal tissue functional synapses will actually develop at the locations of candidate synapses depends on other factors as well. When we use the word synapse in the following, it is meant to mean candidate synapse. The method is based on proximity and crossing of axonal and dendritic line pieces (van Pelt et al., 2010). By varying the positions of the somata of the pre- and post-synaptic neurons, one can obtain the number of synaptic contacts as a function of neuron positions. Repeating this process for many neuron pairs of a population of reconstructed neurons yields a statistical estimate of the number of synaptic contacts vs. soma positions. From these outcomes, one can also derive an estimate of the connection probability (the probability that an arbitrary neuron pair is connected, i.e., has at least one synaptic contact) as well as the mean number of synaptic contacts per connected neuron pair.

The question whether connectivity can also be derived from the overlap of dendritic and axonal density fields has been addressed by Liley and Wright (1994), based on the work of Uttley (1955). They derived an analytical expression for the expected number of synapses between two neurons at given positions. They assumed spherical symmetry in the axonal and dendritic density fields and used exponential decaying radial functions. Their analytical approach in continuous space required smooth density functions. Kalisman et al. (2003) constructed averaged templates of axonal and dendritic fluxes in 3D space (preserving spatial and directional information) for calculating the expected number of contacts. They found a good agreement with the actual number of autapses in reconstructed rat cortical layer 5 pyramidal neurons. Stepanyants and Chklovskii (2005) calculated neurite segment length density functions from reconstructed neurons and applied the formalism of Liley and Wright (1994) to study the relation between neurogeometry and potential synaptic connectivity. In order to obtain spatially smooth density fields, they convolved the skeleton densities with a Gaussian function with a typical standard deviation of 10–30 μm. Recently, McAssey et al. (in revision) used the Liley and Wright method to investigate the propagation of individual neuron variability via the density fields into variability in the estimated number of contacts. Using sets of generated neuron morphologies of different sizes, they showed how the standard deviation in the estimated number of contacts decreases with increasing size of the data set used for calculating the density fields. Instead of deriving connectivity from density fields, Cuntz (2012) followed the reverse way by using a minimal spanning tree approach to derive the dendritic density fields from the spatial distribution of contacts points between the neurons.

Important for the validity of the methodology developed for deriving connectivity from density fields is that the estimated connectivity from overlapping density fields is consistent with the connectivity derived from the actual arborizations. To our knowledge such a rigorous validation has never been carried out before.

The objectives of this paper are (i) to derive connectivity from overlapping density fields using our recently developed criterion for the formation of synaptic contacts (van Pelt et al., 2010); (ii) to develop a method that is also applicable for highly irregular density fields (such as those of individual neurons) and that is thus not dependent on any smoothness requirement of the density fields; (iii) to validate the neuronal connectivity estimates from the overlapping density fields with the actual connectivity derived from mutually innervating axonal and dendritic arborizations.

The method developed here is based on a discretization of space by a grid of voxels of a given size (here set to 1 μm), with each voxel having a certain dendritic and/or axonal mass density. These densities are then used to calculate the probabilities of finding axonal and dendritic line pieces in the voxels. Assuming uniform random orientations of these line pieces in each voxel, we then apply the above mentioned proximity/crossing criterion to axonal and dendritic line pieces (van Pelt et al., 2010) in the same or within different voxels. The connectivity measures between two neurons at given positions are obtained by evaluating all voxel pairs of the axonal and dendritic density fields. The new method is used to make predictions of the expected number of contacts, the connection probability between a pre-synaptic and post-synaptic neuron, and the number of contacts between a connected pre- and post-synaptic neuron pair, using their density fields. In addition, the mean connection probability and the Euclidean distances of synapses to their pre- and post-synaptic somata are estimated in a given network of neurons represented by their density fields.

The data set of neuronal arborizations used for the calculation of the density fields and the actual connectivity between the individual neuronal arborizations (validation) was obtained using our simulator NETMORPH (Koene et al., 2009). A number of 50 random neuron morphologies were generated with growth parameters optimized on a set of rat cortical L2/3 pyramidal neurons, reconstructed by Svoboda (Shepherd and Svoboda, 2005) and made available by the NeuroMorpho.org data base (Ascoli, 2006).

### Summary of findings

An exact expression was derived for the expected number of contacts between two neurons based on the overlap of their axonal and dendritic density fields. This density-field based estimate of the number of contacts turned out to be fully consistent with the number of contacts calculated directly from the actual arborizations. The method is applicable to any arbitrary filling of space with density values, thus also to “fields” obtained from single dendritic or axonal arborizations. No assumptions were needed for the “smoothness” of the density fields. A significant reduction in computational load was achieved when local uniformity of axonal densities in the neighborhood of dendritic densities could be assumed. This approximated expression was consistent with the expression derived by Liley and Wright (1994), using analytical methods. The accuracy of the approximated expression was quantified. Our attempt to estimate the connection probability and the expected number of contacts per connection (connected neuron pair) from the density fields failed because the fields do not carry anymore the underlying correlative structure in the spatial distribution of arbors and synapses. Using empirical mapping functions, however, we could well estimate both connectivity measures from the expected number of contacts. For a network of spatially distributed neurons the average connection probabilities between neuron pairs vs. their intersoma distance were calculated from their population mean density fields. We showed how Euclidean distances of synapses to their pre- and post-synaptic somata can be estimated from the density fields, and how these distances for a centrally located neuron in a network depend on the spatial distribution of the other neurons.

The paper is organized as follows. The Materials and Methods section gives a brief summary of the developed methodology; the developed methodology is fully described in the Appendix (see Supplementary Material). The Results section includes an application part with the estimation of connectivity measures between two neurons based on their density fields, a validation part in which the density-field estimates are compared with the estimates based on the original arborizations, and an application part with the estimation of averaged connectivities between neurons in a network. The findings are discussed in the Discussion section.

## Materials and methods

### Axonal and dendritic mass distributions in a spatial grid of voxels

**Figure 1:**
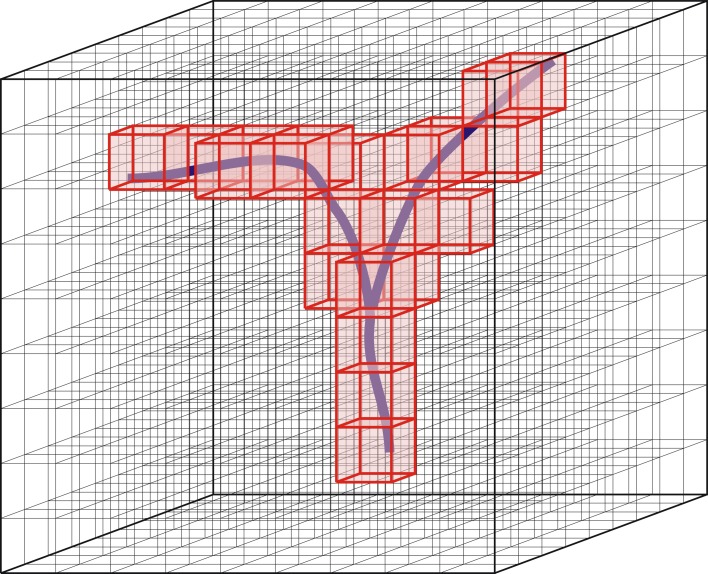
**Discretization of space by means of a three-dimensional grid of voxels**. Red, voxels occupied by the branching structure.

Axonal and dendritic arborizations innervate space in a manner that is determined by their morphological characteristics. Like the morphology of neurons, the spatial innervation patterns of neurons may vary considerably between neurons. To quantify these spatial patterns, we discretize space by a cubic three-dimensional grid, with volume elements (voxels) of size *s_v_* and volume *s*^3^*_v_* μm^3^ (Figure 1).

A single arborization will intersect only a fraction of the voxels in the 3D grid, and within each such voxel it will do so with a certain “mass.” “Mass” in this context refers to the volume or to the length of the arbor structure. In this study we will use the length of the part of the arborization that lies in the voxel, thus ignoring the diameters of the arborizations. For a large number of arborizations aligned according to their somata, many more voxels will be intersected depending on the variability of the arborizations. The summed “mass” per voxel is then a measure for the total mass of the population of arborizations at that location in space. Dividing the summed “mass” per voxel by the number of arborizations gives an estimate for the population mean mass *m_v_* of a single arborization per voxel, or for its density ρ in the case of a unit voxel (*s_v_* = 1 μm). Voxel densities, calculated separately for axonal and dendritic arborizations, result in so-called (population mean) axonal and dendritic density fields. The mass per voxel is then obtained via

(1) $$m_{v}=\rho \times s_{v}^{3}$$

indicating the expected length of an axonal or dendritic arborization in that particular voxel.

### Scale of the 3D grid

The scale of the grid is defined by the size of the individual voxels *s_v_*. Evidently, this size determines the level of fine structure that is preserved in the density fields. Neuronal branches contain branch points and their branches may be curved. Coarse grid scales do not capture these finer details and integrate all length within a voxel. Finer grid scales increasingly capture more linear parts of the branches. A voxel size of 1 μm is considered to be appropriate in capturing the branching structure in all its relevant details. In addition, the intersections of the branches with voxels of this size can be expected to deviate little from straight lines. For fine grid scales, single axonal or dendritic trees will intersect only a small fraction of the total number of voxels. A large number of trees is therefore needed to obtain statistically sufficiently stable density fields of axonal and dendritic arbors.

### Estimation of density fields

A dendritic arbor of a cortical L2/3 pyramidal neuron may fill voxels up to distances of about 400 μm from the soma. With a 1 μm voxel size, there are already 4π^*^400^2^ = 2.010.619 voxels at that distance in 3D space (i.e., the surface of the sphere with a radius of 400 μm). When an individual dendrite reaches such distances with one branch, then only single voxels are intersected at these distances. If one wants a population sum with all voxels at that distance intersected by at least one branch, a total number of about 2^*^10^6^ dendrites is needed. To obtain stable statistical averages per voxel, one needs a multitude of this number, say at least 20 ^*^ 10^6^ dendrites. Axonal fields extend over larger distances of, say, 1000 μm for local arborizations. The number of voxels at this distance from the soma is 4π ^*^ 1000^2^ = 12.566.371 and for stable density field estimates in peripheral areas one needs a number of at least 1.2^*^10^8^ axonal arborizations. Evidently, these are unrealistically high numbers if experimental reconstructed neurons need to be used for building density fields. Neural simulators could possibly do the job, but the numbers are still huge.

The estimation of (smooth) density fields becomes more tractable when the density fields can be assumed to have some symmetry. For instance, if the arborizations invade space without any preferred direction, then spherical symmetry may be assumed. Under these conditions it is sufficient to have a stable estimate of the radial distribution of dendritic mass *M_d_*(r) and axonal mass *M_a_*(r) vs. distance *r* from the soma. The spatial densities ρ per unit volume are then obtained via

(2) $$\rho_{d}(r)=\frac{M_{d}(r)}{4\pi r^{2}}\text{ and }\rho_{a}(r)=\frac{M_{a}(r)}{4\pi r^{2}}.$$

When spherical symmetry cannot be assumed, the arborization may show axial symmetry around a central axis (i.e., being invariant for rotations around the axis). Axial symmetry may be present in cortical pyramidal neurons, with the apical dendritic main stem as the axis of symmetry. Axial symmetry was implicitly assumed in the so-called fan-in projection method by Glaser and McMullen (1984). With axial symmetry it is sufficient to have stable estimates of the mass distribution at different heights *z* and distances *r_p_* perpendicular to the central axis, *M_d_*(*z, r_p_*) and *M_a_*(*z, r_p_*). The spatial densities per unit volume are then obtained via

(3) $$\rho_{d}(z,r_{p})=\frac{M_{d}(z,r_{p})}{2\pi r_{p}}\text{ and }\rho_{a}(z,r_{p})=\frac{M_{a}(z,r_{p})}{2\pi r_{p}}.$$

The estimation of density fields becomes even more tractable without any requirement on smoothness or complete filling of space. In this study 50 neurons are used to construct a population mean axonal and dendritic density field.

### Connectivity and axonal and dendritic density fields

Axons can make synaptic connections with dendrites when their branches are sufficiently close to each other (Peters, 1979). Given reconstructed axonal and dendritic arborizations one can search the whole space for locations of sufficient proximity. With arborizations approximated by series of line pieces, one needs to test all combinations of axonal and dendritic line pieces. An algorithm for such a search has recently been developed by van Pelt et al. (2010). The algorithm is based on the requirement that pairs of axonal and dendritic line pieces cross with a crossing distance smaller than a given criterion distance. In density fields, however, the individual branch structure is lost and replaced by the probability of a finding a certain mass in the individual voxels. The question then becomes how these densities can be used in estimating the connectivity between axons and dendrites. We propose an answer to this question by the following method.

#### Voxel mean intersection length, densities, and hit probabilities

A line intersecting a voxel has intersecting points with two voxel planes. The line piece between these intersecting points, called the intersecting line piece (or intersection), has a certain length *l*_int_. For a voxel of size *s*, *l*_int_ can be as small as 0 μm when the line is intersecting a corner of the voxel and as long as the diagonal in the voxel, thus having a range of $l_{\text{int}}(s)\in \left[0,s\sqrt{3}\right]\mu m$. Intersecting a voxel of size *s* by a large number of randomly oriented lines gives a characteristic distribution of intersection lengths (see Appendix section A1) with a mean of

(4) $$\bar{l_{\text{int}}(s)}=C\times s,\text{with }C=0.66653,$$

and a standard deviation of

(5) $$sd\left(l_{\text{int}}(s)\right)=0.39156\times s.$$

When a randomly oriented line is drawn in a space larger than the voxel, the line may or may not intersect the voxel; that is, in a statistical sense, the voxel will be hit with a certain probability *p*^hit^_voxel_(*s*). When there are *N* randomly oriented lines in that space, the voxel will be hit by an expected number of *E*{*n*^hit^*_v_*} = N × *p*^hit^*_v_*(*s*). The total length of the intersecting line pieces *L*^tot^_int_(*s*) (total mass) then becomes

(6) $$L_{\text{int}}^{\text{tot}}(s)=E\left\{n_{v}^{\text{hit}}\right\}\times \bar{l_{\text{int}}(s)}=C\times s\times E\left\{n_{v}^{\text{hit}}\right\}.$$

Rewriting this equation as

(7) $$E\left\{n_{v}^{\text{hit}}\right\}=\frac{L_{\text{int}}^{tot}(s)}{C\times s}$$

gives us the expected number of intersecting line pieces in a voxel, expressed in terms of the total “mass” in the voxel and the mean intersection length. When the probability of hitting a voxel is very low, this equation applies to the hit probability itself with

(8) $$p_{v}^{\text{hit}}(s)≅\frac{L_{\text{int}}^{\text{tot}}(s)}{C\times s}$$

which gives us the probability that a voxel is hit by a random line in the surrounding space, expressed in terms of the total “mass” in the voxel and the mean intersection length. Let the density ρ denotes the mass per unit voxel (i.e., with *s* = 1 μm), then the mass per voxel of size *s* becomes ρ × *s*^3^. Dendritic mass *m_vd_* and axonal mass *m_va_* in a voxel *v* can now be related to the probability *p*^hit^*_vd_* that a voxel *v* is intersected by a dendritic branch and the probability *p*^hit^*_va_* that the voxel is intersected by an axonal branch, respectively:

(9) $$\begin{array}{l} p_{vd}^{\text{hit}}(s)≅\frac{m_{vd}}{C\times s}=\frac{\rho_{vd}\times s^{3}}{C\times s}=\frac{\rho_{vd}\times s^{2}}{C}\\ \text{ and}\\ p_{va}^{\text{hit}}(s)≅\frac{\rho_{va}\times s^{2}}{C}. \end{array}$$

(see also Appendix section A2).

#### Crossing line pieces, crossing probabilities, and crossing distances

An estimate can now be made of the connectivity between axonal and dendritic arborizations when they are expressed in terms of density fields (see also Appendix section A3). Two infinite lines in space are at their shortest distance at the site where they are crossing. At this site a connection line can be drawn orthogonal to both infinite lines, with a length called crossing distance. Although two infinite lines will cross with certainty (except when they are parallel or coincide), two line pieces with finite length may or may not cross in space. This principle is used for defining possible unique synaptic locations between dendritic and axonal arborizations, with the additional requirement that in the case of crossing the crossing distance should not be larger than a given distance criterion (van Pelt et al., 2010).

The crossing of random intersections in a single voxel or in different voxels is described in Appendix section A3. The results are briefly summarized here. The probability *p*^cross^ that a pair of random line pieces in a single voxel cross is equal to

(10) $$p^{\text{cross}}=0.3133,$$

which is independent of the size of the voxel. In contrast, crossing distances between crossing line pieces in a single voxel do scale linearly with the size *s* of the voxel and are given by their mean and standard deviation

(11) $$\bar{d^{\text{cross}}}=0.334\times s;\text{and }d^{\text{cross}}(sd)=0.256\times s,$$

(**Figure A5**). For a pair of voxels *v* and *w* at a given distance *d_v, w_* from each other, the crossing probability of random line pieces in both voxels is dependent on the voxel distance, as shown in the graph of **Figure A6**. A best fit through the data points was given by Equation A16

(12) $$p_{v,w}^{\text{cross}}\left(d_{v,w}\geq 1\right)≅0.04467\times {\left(d_{v,w}-0.1966\right)}^{-1.8264}.$$

#### Conditional crossing probabilities

When a distance criterion of δ μ m is set to the crossing distance between crossing line pieces the conditional crossing probability

(13) $$p_{v,w}^{\text{cross}}(s,d_{v,w}|\delta )$$

becomes dependent on δ and on the size of the voxel (see also Appendix section A4). For two random lines in a single voxel the conditional crossing probability *p*^cross^*_v, v_*(s|δ) is shown in **Figure A7** of Appendix section A4.1. For two random lines in different voxels *v* and *w* at a distance *d_v, w_* from each other, the conditional crossing probability *p*^cross^*_v, w_*(*s, d_v, w_*|δ) is shown in **Figure A8** of Appendix section A4.2 (for the unconditional values, see **Figure A6** of the Appendix section A3.2). The figures illustrate how the crossing probability decreases with increasing distance between the voxels particularly when this distance is near the criterion value (**Figure A8**). Note that the distance between voxels is taken as the distance between corresponding voxel corners (or centra). The crossing distances of crossing line pieces in voxel pairs are in the range of $\left[d_{v,w}-s\sqrt{3};d_{v,w}+s\sqrt{3}\right]$.

(14) $$\begin{array}{l} p_{v,w}^{\text{cross}}\left(s,d_{v,w}\gg \delta |\delta \right)=0,\\ p_{v,w}^{\text{cross}}\left(s,d_{v,w}\ll \delta |\delta \right)=p_{v,w}^{\text{cross}}\left(s,d_{v,w}\right),\\ p_{v,w}^{\text{cross}}\left(s,d_{v,w}≅\delta |\delta \right)<p_{v,w}^{\text{cross}}\left(s,d_{v,w}\right). \end{array}$$

#### Density-weighted conditional crossing probabilities

In the foregoing the crossing probabilities were determined on the basis of the presence of a random line piece in a voxel. When the presence of a line piece is a stochastic event then the crossing probabilities need to be multiplied with the probabilities that the line pieces are present (see Appendix section A5). In that case, the crossing probability of line pieces in two voxels *v* and *w* at a given distance *d_v, w_* from each other is given by

(15) $$\begin{array}{l} p_{v,w}^{\text{cross}}\left(s,d_{v,w}|\delta \right)\times p_{v}^{\text{hit}}\times p_{w}^{\text{hit}}=p_{v,w}^{\text{cross}}\left(s,d_{v,w}|\delta \right)\times \frac{\rho_{v}\times s^{2}}{C}\times \frac{\rho_{w}\times s^{2}}{C}\\ \;=\frac{s^{4}}{C^{2}}\times p_{v,w}^{\text{cross}}\left(s,d_{v,w}|\delta \right)\times \rho_{v}\times \rho_{w}.\;\; \end{array}$$

In the overlap area of a dendritic density field *D* and an axonal density field *A*, each voxel has a dendritic and an axonal mass that determines the probability of finding a dendritic or an axonal line piece in these voxels, which is dependent on the size of the voxels. The probability that a dendritic line piece in voxel *v* and an axonal line piece in voxel *w* cross is now given by

(16) $$\frac{s^{4}}{C^{2}}\times p_{v,w}^{\text{cross}}\left(s,d_{v,w}|\delta \right)\times \rho_{vD}\times \rho_{wA},$$

with ρ*_vD_* the dendritic density in voxel *v* and ρ *_wA_* the axonal density in voxel *w*.

#### Expected number of synapses in overlapping axonal and dendritic density fields

The expected number of crossing line pieces of the axonal and the dendritic field in the overlap area can now be obtained by calculating the expected number of crossing axonal and dendritic line pieces in all the pairs of axon and dendrite voxels in the overlap area that meet the distance criterion.

(17) $$\begin{array}{l} E\left\{n_{D,A}^{\text{cross}}|\delta \right\}=\frac{s^{4}}{C^{2}}\times \sum_{v}^{\text{space}}\sum_{w}^{\text{space}}p_{v,w}^{\text{cross}}\left(s,d_{v,w}|\delta \right)\times \rho_{vD}\times \rho_{wA}\\ \;=\frac{s^{4}}{C^{2}}\times \sum_{v}^{\text{space}}\rho_{vD}\times \sum_{w}^{\text{space}}\rho_{wA}\times p_{v,w}^{\text{cross}}(s,d_{v,w}|\delta ).\; \end{array}$$

Assuming that a synaptic connection may be present at locations where axonal and dendritic line pieces cross each other at sufficient small crossing distances, we now have an expression for the expected number of synaptic contacts in the overlap area of axonal and dendritic density fields, given by

(18) $$E\left\{n_{D,\;A}^{\text{synapse}}|\delta \right\}=E\left\{n_{D,\;A}^{\text{cross}}|\delta \right\}.$$

The double summation in Equation 17 runs over all voxel pairs (*v*, *w*) in the given space. However, for each dendritic voxel *v* only the axonal voxels *w* within the criterion distance δ contribute to the sum. The second summation over the axonal voxels *w* can therefore be restricted to the ones in the local environment *v*_env_ (see Equation A23) of the dendritic voxel *v*:

(19) $$E\left\{n_{D,\;A}^{\text{synapse}}|\delta \right\}=\frac{s^{4}}{C^{2}}\times \sum_{v}^{\text{space}}\rho_{vD}\times \sum_{w}^{v_{\text{env}}}\rho_{wA}\times p_{v,w}^{\text{cross}}(s,d_{v,w}|\delta ).$$

#### Approximation of the expected number of synapses—local uniformity in axonal densities

If it can be assumed that the axonal densities ρ*_wA_* in the local environment of a dendritic voxel *v* are not very different from the axonal density ρ*_vA_* in voxel *v* itself, Equation 19 can be simplified into

(20) $$E\left\{n_{D,\;A}^{\text{synapse}}|\delta \right\}≅\frac{s^{4}}{C^{2}}\times \sum_{v}^{\text{space}}\rho_{vD}\times \rho_{vA}\times \sum_{w}^{v_{\text{env}}}p_{v,w}^{\text{cross}}(s,d_{v,w}|\delta ).$$

The second summation now runs over all voxels in the local environment of a given voxel *v* but does not depend on the position of voxel *v* anymore. The outcome that we will call the *local environment crossing factor f*^env^(*s*, δ) now becomes a fixed number that is only dependent on the size of the voxels *s* and the distance criterion δ (see Appendix section A5.2):

(21) $$f^{\text{env}}(s,\delta )=\sum_{w}^{v_{\text{env}}}p_{v,w}^{\text{cross}}\left(s,d_{v,w}|\delta \right).$$

The values of the *local environment crossing factor f*^env^(*s*, δ) are shown in **Table A1** of Appendix section A5.2.1. The *local environment crossing factor f*^env^(*s*, δ) can be approximated by a linear dependence on the criterion δ as (for *s* = 1) *f*^env^(*s* = 1, δ)≅ 0.69822 × δ (Equation A38). Then, Equation 20 simplifies into

(22) $$E\left\{n_{D,\;A}^{\text{synapse}}|s=1,\delta \right\}≅1.572\times \delta \times \sum_{v}^{\text{space}}\rho_{vD}\times \rho_{vA}=1.572\times \delta \times I_{DA},$$

with *I_DA_* denoting the overlap sum $I_{DA}=\sum_{v}^{\text{space}}\rho_{vD}\times \rho_{vA}$.

#### Connection probability and number of contacts per connection (connected neuron pair)

The *connection probability* of two neurons denotes the probability that they are connected, i.e., have at least one synaptic contact. The question whether and how the connection probability for a neuron pair can be estimated from their population mean density fields, can be answered as follows: Let *E*{*n*^syn^*_v_*} denotes the expected number of synapses in voxel *v*. Because the voxel size is small, this expected number will be much smaller than one and can be interpreted as the probability *p*^syn^*_v_* of finding a synapse in that voxel. The probability of no-synapse in that voxel *p*^nosyn^*_v_* is then given by *p*^nosyn^*_v_* = 1 − *p*^syn^*_v_*. The product of the no-synapse probabilities of all voxels in area *A*, assuming independency, then yields the probability of no-synapse in the overlap space, *p*^nosyn^*A* = ∏*_i_*(1 − *p*^syn^*_vi_*). The connection probability *p*^con^*_A_*, i.e., the probability of at least one contact in the overlap space, is then given by *p*^con^*_A_* = 1 − *p*^nosyn^*_A_*.

A basic assumption in this approach is that the synapse probabilities of all the voxels are independent of each other. As will be shown in the Results section, this approach gave inconsistent outcomes, indicating that the basic assumption of independency is not justified. Alternatively, the connection probability was estimated from the expected number of contacts by using a mapping function derived from the connectivity between the actual arborizations. Also for the estimation of the *number of contacts per connected neuron pair* from the expected number of contacts a mapping function was used that was derived from the connectivity between the actual arborizations.

#### Euclidean distances of synapses to their pre- and post-synaptic somata

Euclidean distance distributions of synapses to their pre- and post-synaptic somata can also be obtained from the overlapping density fields. For a given neuron pair the probability of finding a synaptic contact is calculated in each voxel of the overlap space. With the Euclidean distance of this voxel to the pre- and post-synaptic somata, the probability of the synaptic contact is then accumulated to the pre- and post-synaptic Euclidean distance probability distribution, respectively. Summing over all voxels then yields the distance distributions for a single neuron pair. In an area with many neurons this procedure must be repeated for all neurons pairs. The final pre- and post-synaptic Euclidean distance distributions, averaged over all neuron pairs, thus depend on the number and positions of all the somata.

## Results

### Estimation of the connectivity between an axonal and a dendritic neuron using population mean density fields

**Figure 2:**
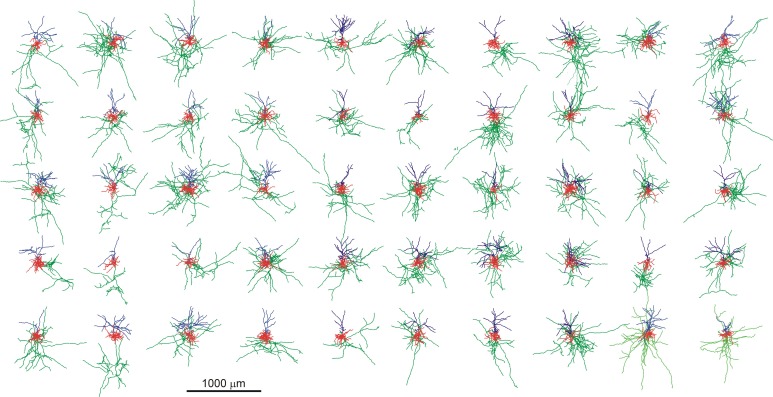
**Display of the set of 50 random neuronal morphologies with their axonal (green), basal (red), and apical (blue) dendritic arborizations, generated with the NETMORPH simulator using a parameter set optimized on a set of rat cortical layer 2/3 pyramidal neurons from the NeuroMorpho.org database**. The neurons are aligned according to their apical dendrites.

For the application of the method the morphologies of a number of 50 neurons were generated with the simulator NETMORPH, using a parameter set optimized on a set of rat layer 2/3 pyramidal cells obtained from the Svoboda data set in the NeuroMorpho.org data base (Figure 2).

### Density fields with axial symmetry

**Figure 3:**
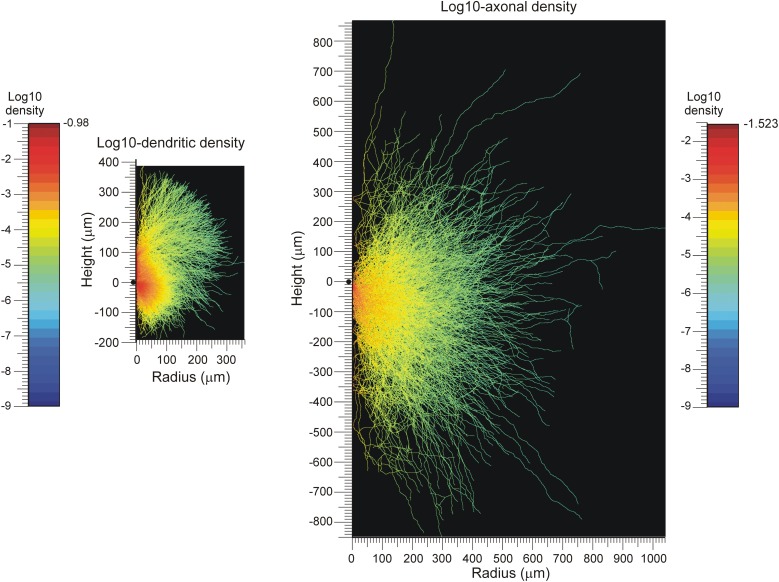
**Population mean density distributions of (left) dendrites and (right) axons of 50 aligned neurons, plotted as function of the axial (height) and radial positions**. The color-coded log10-density scales run from the values indicated at the left of the color bars. The solid dots along the height axes indicate the position of the cell body. Note that a number of −9 was assigned to voxels whenever their original density was zero.

An example of density field calculations based on axial symmetry is given in Figure 3. Assuming that the axial symmetry axis coincides with the apical main stem of the neuron, we calculated the axonal and dendritic mass of 50 NETMORPH-generated neurons as a function of the position along the symmetry axis (height, also referred to as *Z*-axis) and the radial distance (radius, i.e., orthogonal distance to the symmetry axis. To this end, each neuron was first soma-centered at the origin and aligned according to its apical main stem, and then “sliced” into layers of 1 micron thick. Subsequently, the axonal and dendritic intersections per layer were analyzed for their radial mass distribution. The axonal and dendritic density fields are calculated by dividing the “mass” at a given height and radius *r* from the symmetry axis by the perimeter (2π *r*) of the circle with radius *r*, under the assumption that the mass is distributed uniformly over the circle centered at the symmetry axis. These density fields are shown in Figure 3. Because of the large perimeters of the circles, the densities decrease rapidly with increasing radius down to very low levels at large radial distances, as shown in the logarithmic plot for the density field. These plots also show the ranges over which the axons and dendrites send their branches. The population mean density fields clearly show the non-smoothness due to the isolated branches in remote areas from the soma center.

**Figure 4:**
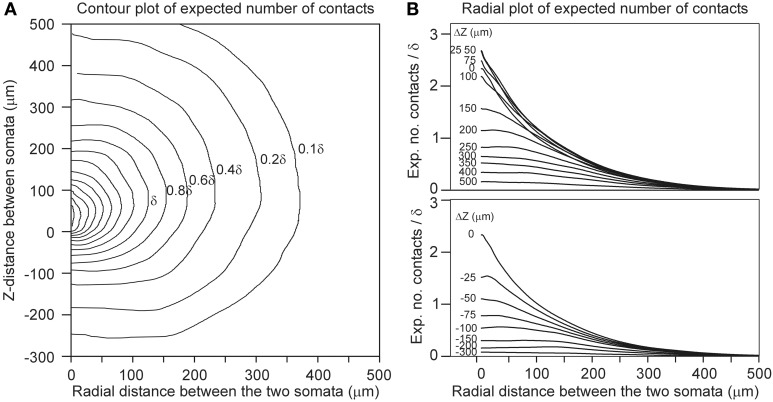
**Expected number of contacts between two neurons shown in (A) contour and (B) axial-radial plots**. The neurons are aligned according to their apical main stem. In all the plots the dendritic neuron is soma-centered at the origin. In **(A)** the position of the soma of the axonal neuron is given by the coordinate axes in the plot. The contours are labeled by the respective values of the expected number of contacts (as a multiple of the criterion value δ, the inner contours maintain the stepwise increase of 0.2δ). In **(B)** the radial position of the axonal soma is given by the abscissa coordinate, while each curve is labeled with the positive (upper panel) and negative (lower panel) displacement along the *Z*-axis (Δ*Z*) of the axonal soma relative to the dendritic soma. The ordinate scale is normalized for δ = 1 μ m.

Finally, for a given spatial positioning of the two cell bodies, the overlap sum *I_DA_* (Equation 22) of the axonal and dendritic density fields was determined by calculating for each voxel the density product of both fields and summing these products over all voxels in the overlap area. Subsequently, Equation 22 is used to calculate the expected number of contacts between both neurons for various values of the proximity criterion δ. The outcomes are given in Figure 4 as contour plots (panel A) and axial-radial curves (panel B), which show how the expected number of synaptic contacts decrease monotonically with increasing distance between the cell bodies. Note that the expected number of contacts has its maximum when the pre-synaptic neuron is positioned about 50 μm above the post-synaptic neuron.

### Validation of the density-field estimated number of contacts between two neurons

**Figure 5:**
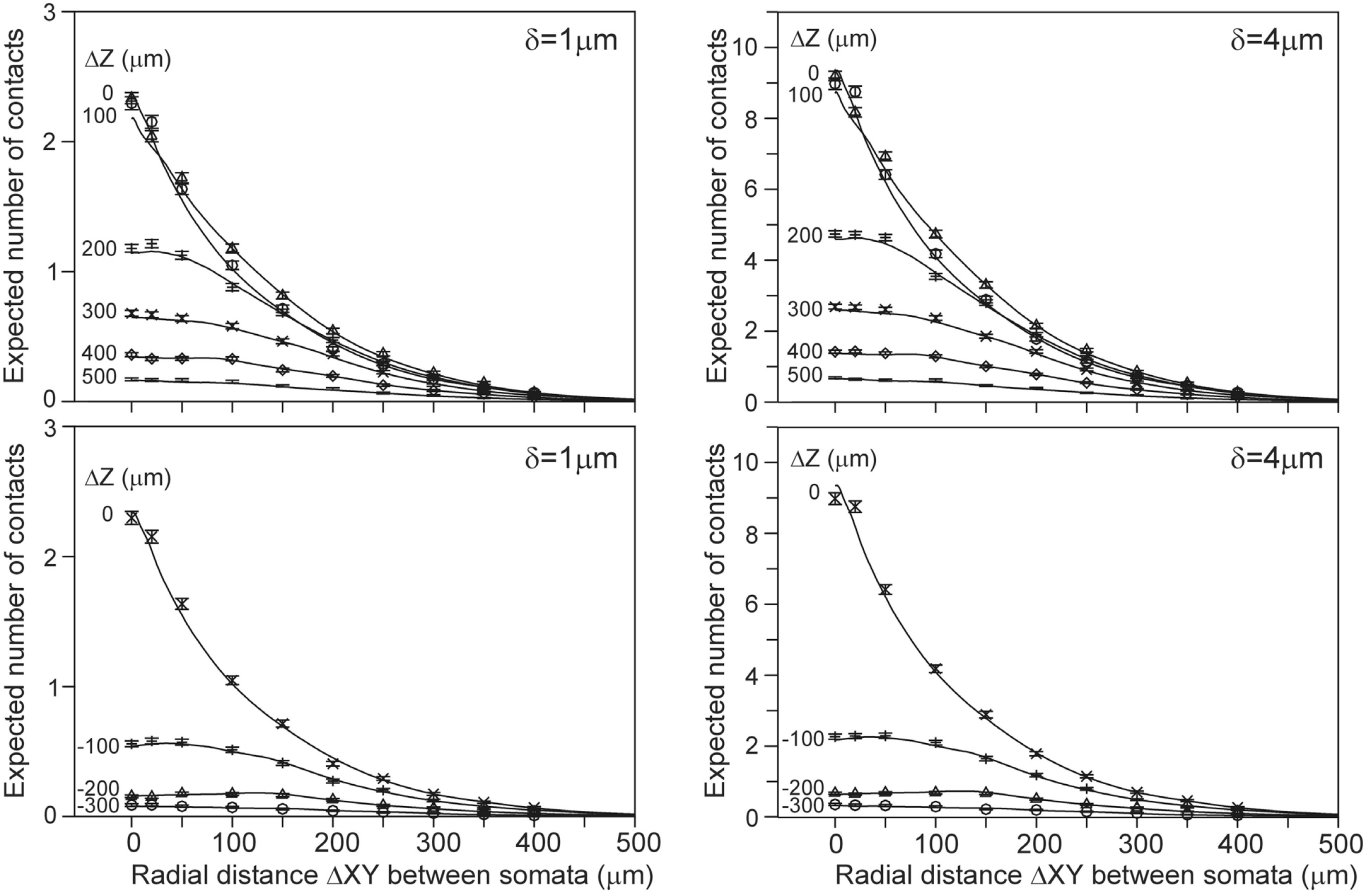
**Comparison of the expected number of contacts predicted by the population mean density-field approach (solid curves), and obtained directly from the axonal and dendritic arbors of the aligned neurons [individual data points with mn(sem) values]**. Each curve is labeled with the positive (**Upper panels**) and negative (**Lower panels**) displacement along the *Z*-axis (Δ*Z*) of the axonal soma relative to the dendritic soma. Shown are the validations for criterion values of δ = 1 (**Left column**) and δ = 4 (**Right column**). The validations for δ = 2 and δ = 3 showed a similar agreement between density-based and arbor-based calculations (not shown in figure).

The number of contacts estimated from overlapping axonal and dendritic density fields is validated by comparison with the number of contacts between the actual 3D arborizations of the same data set of simulated neurons. The actual number of contacts was determined for all the 50^*^49 = 2450 neuron pairs with the soma of the dendritic neuron centered at the origin and the soma of the axonal neuron positioned at a given axial and radial distance. The number of contacts was determined by assessing, for all the pairs of dendritic and axonal line pieces, whether they were crossing and whether the crossing distance was smaller than or equal to the given proximity criterion (van Pelt et al., 2010). The mean number of contacts for all the neuron pairs, and the mean number of contacts for all the connected neuron pairs, were determined for a number of different axial and radial positions of the axonal cell bodies. The results are shown in Figure 5. The solid curves indicate the expected number of contacts from the density fields; these curves are identical to the ones in Figure 4. The individual data points show the mean and standard error in the mean (sem) (*n* = 2450) of the number of contacts actually determined from the overlapping axonal and dendritic arbors between all neuron pairs. An excellent agreement was found between the density-based expectations and the arbor-based calculations, even within the small standard error values. A similar agreement was found for the distance criteria δ = 2 and δ = 3 (not shown in Figure 5). Although the actual number of contacts is highly variable between neuron pairs, as reflected in the standard deviation in the distribution of data points (Figure 10), it is because of the large number of 2450 data points that the sem values become very small. This agreement thus validates the density field approach for estimating the number of contacts between neuron pairs.

### Estimation of the connection probability from the expected number of contacts

**Figure 6:**
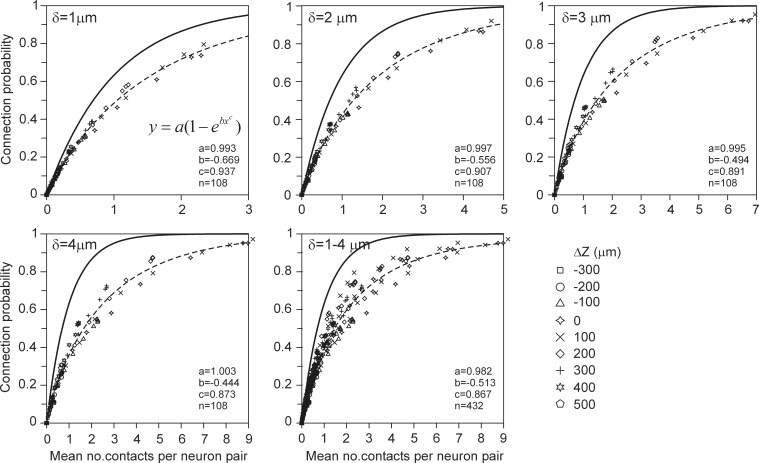
**Scattergram of the mean connection probability vs. the mean number of contacts (obtained from the actual arbors of all the 2450 neuron pairs in the validation set)**. Each panel is labeled by the used distance criterion δ and includes the theoretical mapping function (solid line; see also **Figure A13**), a best-fitting regression function (dashed line) through the data points of the type *f*(*x*) = *a*(1 ‒ *e^bx^c^^*), and the values of the optimized parameters. The data points are labeled by their *z*-shift values (see symbols).

The connection probability between two neurons was calculated from their population mean density fields according to the approach described in the Materials and Methods section. For validation, the connection probability was also calculated from the actual arborizations as the ratio of the number of connected neuron pairs (with at least one contact) and the total number of 2450 neuron pairs. Both approaches turned out to give inconsistent results. The density-field expected values were significantly larger than the arbor-based data points. A generalization of the approach in the Materials and Methods section is further described in Appendix section A7, where it is explained how the connection probability between two neurons can be estimated from the expected number of contacts when independency is assumed for the spatial distribution of synapses. This resulted in a “theoretical” mapping curve, which is shown in **Figure A13** and in Figure 6 (solid curve). The relation between the connection probability and the expected number of contacts, estimated from the population mean density fields, was found to exactly match this theoretical mapping curve. However, the density-field estimated connection probability was inconsistent with the arbor-based connection probability. This thus implicated that the theoretical mapping function was not appropriate. For validation, it was compared with an empirical mapping function, derived from the arbor based calculated number of contacts and connection probability. To this end, for a given spacing of the cell bodies, both the mean number of contacts and the connection probability for all the 2450 neuron pairs were determined from the actual arborizations. By varying the spacing for *x*-shifts of (0, 20, 50, 100,…, 500 μm) and *y*-shifts of (−300, −200,…, 500 μm) one obtains 12^*^9 = 108 data points, as shown in the scatterplots of Figure 6. The actual data points indeed show significantly lower connection probabilities than those predicted by the theoretical curve (upper solid curve). For low number of contacts the data points are very close to but do not exceed the theoretical curve. Apparently, the theoretical curve, derived from the expected number of contacts, provides an upper limit for the connection probability. Figure 6 includes best-fitting regression functions of the type *f*(*x*) = *a*(1 − *e^bx^c^^*) through the data points. The method for calculating the connection probability (see Materials and Methods section) and its generalization in A7 are based on the assumption that the expected number of contacts in the voxels in the overlap space are independent of each other (see also Equation A49). The incorrectness of this assumption is likely caused by the fact that synapses are restricted in their positions to the axonal and dendritic arborizations, which provide an underlying correlative structure to the synapse positions that is not reflected anymore in the density fields.

An explanation for the overestimation of the connection probability can be given by referring to the procedure in Section Connection Probability and Number of Contacts per Connection (Connected Neuron Pair). Because actual synapses are restricted to the arbor subspace they are spatially correlated. In other words, finding an actual synapse implicates a higher probability of finding another actual synapse in that subspace. Similarly, not finding an actual synapse at a given location implicates a high probability to be not at the arbor subspace and also implicates a higher probability of not finding an actual synapse nearby. In the density field approach the probability of finding or not finding a synapse at a given location (voxel) is assumed to be independent of the probability of finding or not finding a synapse elsewhere, respectively. The product of the probabilities of not finding a synapse in the different locations in the overlap area is thus higher in the actual case than in the density field case. Consequently, the probability of at least one contact will be lower in the actual case than in the density field case. Thus, the density field approach overestimates the connection probability between two neurons.

**Figure 7:**
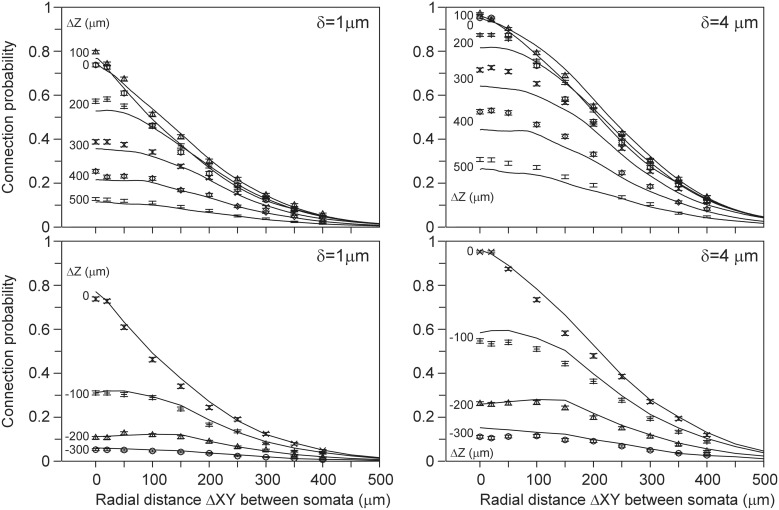
**(Solid lines) Connection probabilities estimated from the expected number of contacts using the best-fitting mapping functions shown in Figure 6**. Each curve is labeled with the positive (**Upper panels**) and negative (**Lower panels**) displacement along the *Z*-axis (Δ*Z*) of the axonal soma relative to the dendritic soma. Individual data points are the arbor-based results. Results are shown for distance criterion values of δ = 1 μ m (**Left column**) and δ = 4 μ m (**Right column**). For δ = 2 μ m and δ = 3 μ m a similar agreement between density-based estimations and arbor-based calculations was obtained (not shown).

Because the connection probability could not be estimated from the density fields, we alternatively estimated it from the (correct) density-field estimated number of contacts using the empirical mapping functions. The results, shown in Figure 7 for distance criterion values of δ = 1 μ m and δ = 4 μ m, are now in good agreement with the validation data, and for several cell positions the expected curves even agree within the sem values of the validation data. However, for other cell-cell positions the validation data lie somewhat above or below the expected curve. Figure 6 shows that the scatter of the validation data around the best-fitting curve is not random but mainly positive or negative for the different cell-cell positions. For instance, the Δ*Z* = 0 data points are lower than the curve, whereas the Δ*Z* = 200 data points lie above the curve. This structure in the variation of the data translates also directly into the deviations shown in Figure 7. The small deviations between the expected and validation data can therefore be explained by the structure in the variations in the validation data, which appeared to depend on the cell-cell positions. Thus, the connection probabilities can be well-estimated from the density-field expected number of contacts using the empirical mapping function.

### Estimation of the expected number of contacts per connected neuron pair

**Figure 8:**
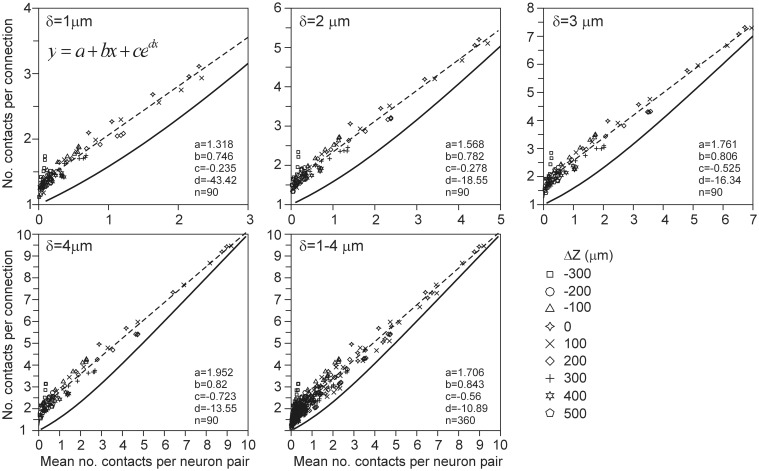
**Scattergram of the mean number of contacts per connection vs. the mean number of contacts (obtained from the arbors of all the 2450 neuron pairs in the validation set)**. Each panel is labeled by the distance criterion δ and includes the theoretical mapping function (solid line and see **Figure A13**), a best-fitting regression function (dashed line) through the data points of the type *f*(*x*) = *a* + *bx* + *ce^dx^*, and the values of the optimized parameters.

The expected number of contacts per connection between two neurons is defined as the mean of the number of contacts in a connected neuron pair, averaged over all the connected neuron pairs in the data set. This number is equal to the ratio of the expected number of contacts and the connection probability (Equation A50 in Appendix section A7). But similarly to the connection probability, the density-field expected values were significantly different from the validation data. These deviations can be seen in Figure 8 by comparing the relationship between the number of contacts per connection vs. the number of contacts as predicted from the density-field approach (thick solid line) and calculated from the actual arborizations (individual data points). The empirical mapping functions (dashed lines in Figure 8) were obtained by regressing the data points with a function of the form *f*(*x*) = *a* + *bx* + *ce^dx^*. The theoretical mapping curve (solid line in Figure 8) is also shown in Appendix section A7 (**Figure A13**).

**Figure 9:**
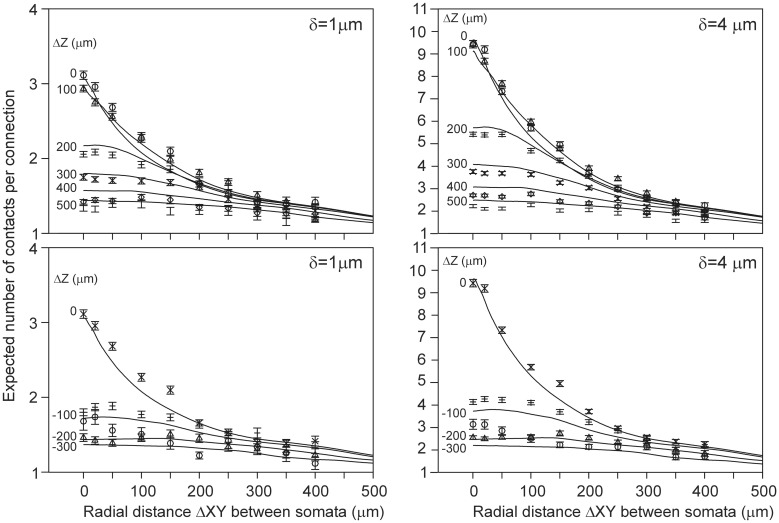
**(Solid lines) Number of contacts in connected neuron pairs, estimated from the expected number of contacts using the best-fitting mapping function shown in Figure 8**. Each curve is labeled with the positive (**Upper panels**) and negative (**Lower panels**) displacement along the *Z*-axis (Δ*Z*) of the axonal soma relative to the dendritic soma. Individual data points are the arbor-based results. Results are shown for distance criterion values of δ = 1 μ m (**Left column**) and δ = 4 μ m (**Right column**). For δ = 2 μ m and δ = 3 μ m a similar agreement between density-based estimations and arbor-based calculations was obtained (not shown).

Because the number of contacts per connection also could not be estimated from the density fields, we alternatively estimated it from the (correct) density-field estimated number of contacts using the empirical mapping functions. The results for δ = 1 μ m and δ = 4 μ m are shown as solid curves in Figure 9. The estimated values appear to be in very good agreement with the validation data for several cell-cell positions, even within the sem values of the data points. But for other cell-cell positions the deviations show the same systematic structure as in the scatterplots of Figure 6, indicating that they originate from the variability structure in the validation data for the different cell-cell positions. Thus, also the number of contacts per connection can be well-estimated from the density-field expected number of contacts using the empirical mapping function.

### Density fields of individual neurons—validation of equation A24

In Equation A24 it was shown that the expected number of contacts obtained from the overlap of population mean density fields is equal to that obtained from the sum of the overlap of individual neuron density fields. To test this equality, we estimated the expected number of contacts in a neuron pair from the overlap between the axonal and dendritic density fields of the individual neurons at given spatial locations by means of the exact expression (A24). Next, the outcomes were averaged over all the 2450 neuron pairs. The calculations were repeated for a range of mutual locations of the neuron pairs. The distributions for the averaged expected number of contacts between individual neuron density fields turned out to match exactly the ones obtained from the population mean density fields as shown in Figure 4. This agreement thus validates Equation A24 and also demonstrates that connectivity estimates based on density fields of individual neurons give consistent results, irrespective of the irregularities of the individual neuron fields.

### Density fields of individual neurons—variability in the connectivity between neurons

**Figure 10:**
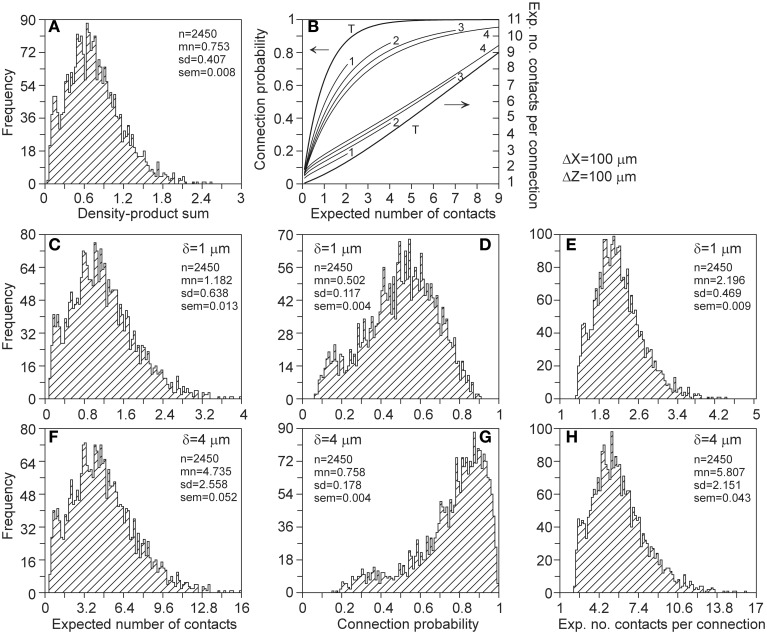
**Distributions of connectivity measures of the 2450 pairs of individual neurons, with the axonal neuron placed at an *x*-shift of 100 μm and a *z*-shift of 100 μm**. Shown are the distributions of **(A)** the density-product values, **(C,F)** the expected number of contacts, **(D,G)** the connection probability, and **(E,H)** the expected number of contacts per connected neuron pair. These measures were calculated for proximity criteria of δ = 1 μ m (2nd row) and δ = 4 μ m (3rd row). The top-right panel **(B)** shows the mapping functions used for calculating the connection probability (left ordinate) and the expected number of contacts per connection (right ordinate) from the expected number of contacts. The curves are labeled with the value of the proximity criterion δ, with T denoting the theoretical mapping curve.

Measures of connectivity between individual neuron pairs show large variations. As illustration, connectivity measures were calculated for all the 2450 neuron pairs, with the axonal neuron placed at an *x*-shift of 100 μm and a *z*-shift of 100 μm relative to the dendritic neuron. Again the exact expression (A24) was used. The distributions of these measures are shown in Figure 10.

### Density fields of individual neurons—validation of the local uniformity assumption in the axonal density in the calculation of the density fields overlap

**Figure 11:**
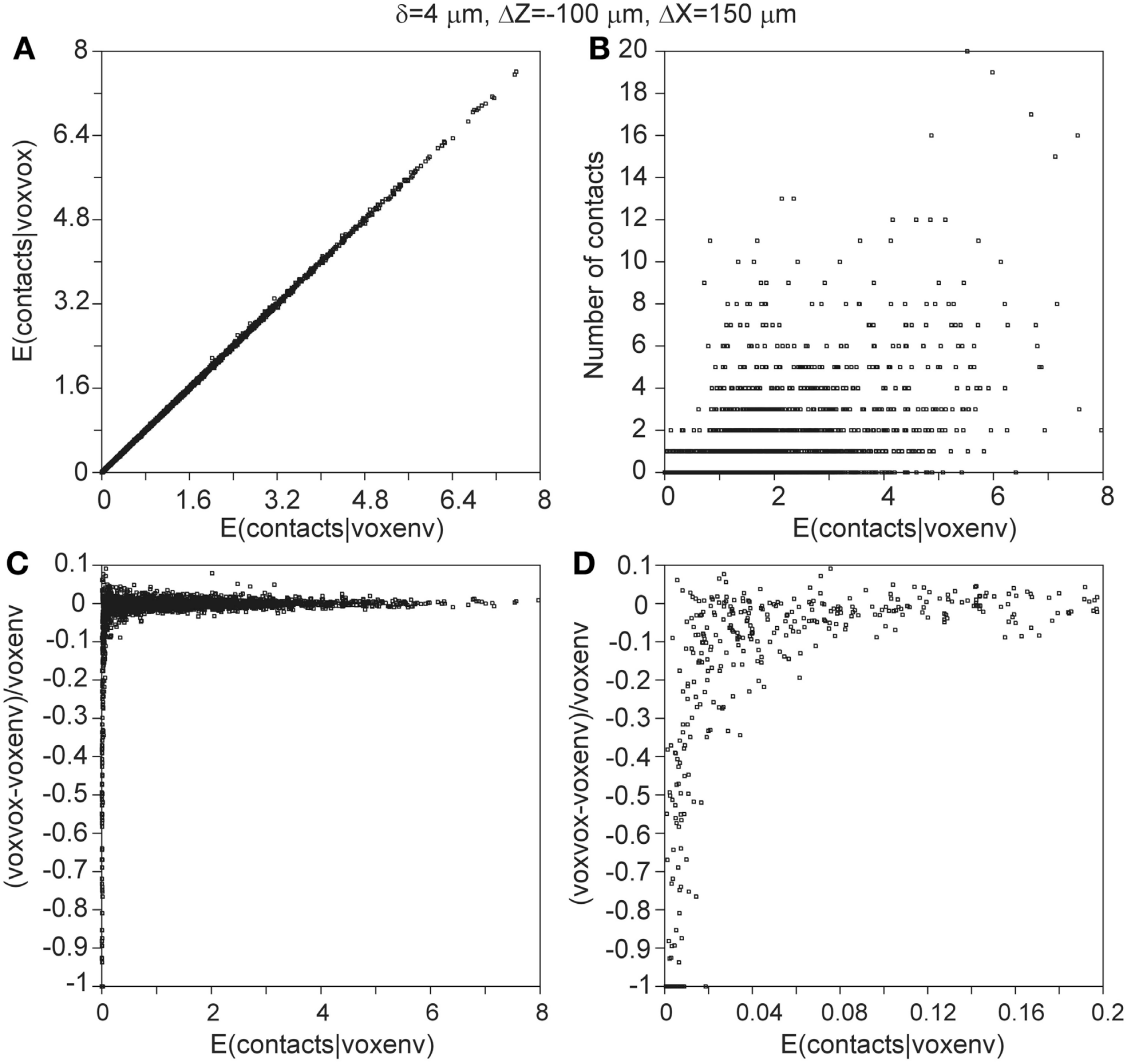
**(A)** Expected number of contacts between two neurons calculated with the approximated voxel-voxel overlap approach (ordinate) vs. the expected number of contacts calculated with the exact voxel-environment overlap approach (abscissa). **(B)** Actual number of contacts between two neurons vs. the expected number of contacts calculated with the voxel-environment overlap approach. Note that many data points are plotted over, but this information is not essential because the figure only aims at illustrating the range of actual values underlying a given expected value. **(C)** Relative difference between expected number of contacts according to the voxel–voxel and voxel-environment approach vs. expected number of contacts according to the voxel-environment approach. **(D)** Similar to **(C)** but with finer abscissa scale.

Thus far, all the calculations involving the population mean density fields used the approximated expression in Equation 22, which is based on the assumption that the axonal densities in the local environment of a dendritic voxel do not differ much from the axonal density in the dendritic voxel itself. For a density field that is calculated as the mean of a large population of neurons, this is a reasonable assumption. For density fields of individual neurons, however, this may not be a good assumption, as the density field then reflects the individual arbors, which are not filling space in a smooth manner. This is also the case when the density field is obtained by spreading arbor mass in an axial symmetric way. Therefore, also the approximated expression of Equation 22 needs to be validated. To this end, the number of contacts between two neurons is calculated using (1) the approximated expression of Equation 22, (2) the exact expression in Equation 19, and (3) the actual contacts points between the arbors themselves. The results for the 2450 neuron pairs, with the axonal soma shifted −100 μm in the Z-direction and 150 μm in the X-direction relative to the dendritic soma, and with δ = 4μ m, are displayed in Figure 11. When the approximated expectations are plotted vs. the exact expectations for all the 2450 neuron pairs, they show a clear diagonal pattern (Figure 11A). When the relative difference between the approximated and the exact expectations are plotted vs. the exact expectations, the data points show a jitter around zero, with larger fluctuations for smaller values of the exact expectations (Figure 11C). For very small values of the exact expectations, the approximated expectations are systematically smaller than the exact expectations (Figure 11D). These findings can be understood by realizing that the approximated expectations are based on the product of axonal and dendritic densities per voxel. In the case of a positive dendritic density but zero axonal density, the product will be zero. For the exact expectation, however, also the axonal densities in the environment of the dendritic voxel contribute to the density product sum, implicating that even when the axonal density in the dendritic voxel is zero its environment may contribute positively. Thus, for small values of the expected number of contacts, the approximated expectation as given by Equation 22 underestimates this number. Figure 11B shows the comparison of the exact expectation of the number of contacts with the actual number of contacts between the overlapping axonal and dendritic arbors of all the 2450 neuron pairs. It is clear from Figure 11B that even if the actual arbors do not have contacts, the expected number of contacts can be positive. Also, for a given value of the expected number of contacts the actual numbers of contacts can range between zero and 20, a range also shown in Figure 10F.

For the expected mean number of contacts, averaged over all the 2450 neuron pairs, with *z*-shift = −100 μm and *x*-shift = 150 μm, the relative differences between the exact and approximated expectations were −0.003% (δ = 1μ m), −0.005% (δ = 2 μ m), 0.037% (δ = 3 μ m) and 0.016% (δ = 4 μ m). Compared over a large number of soma-soma positions, the mean value of the expected number of contacts between two neurons calculated with the exact and the approximated expression (both averaged over all 2450 neuron pairs) showed a relative difference of less than 0.05% (δ = 1 μ m), 0.1% (δ = 2 μ m), 0.2% (δ = 3 μ m), and 0.2% (δ = 4 μ m).

### Network applications of density fields

Thus far, the focus was on using density fields for estimating the connectivity between two neurons at given positions in space (see Figures 5, 7, 9). In a network, however, neurons all take their individual positions. Network connectivity is therefore determined by the mean of the connectivities between all pairs of neurons. Evidently, this network connectivity is highy dependent on the actual positions of the neurons. An example is given in section Estimation of the Connection Probability in Neuronal Networks for the averaged connection probability in a network. Density fields can also be used for estimating the Euclidean distance distributions of synapses to their pre- and post-synaptic somata. The method and example results are explained and discussed in section Estimation of Euclidean Distances of Synapses to their Pre- and Postsynaptic Somata.

#### Estimation of the connection probability in neuronal networks

**Figure 12:**
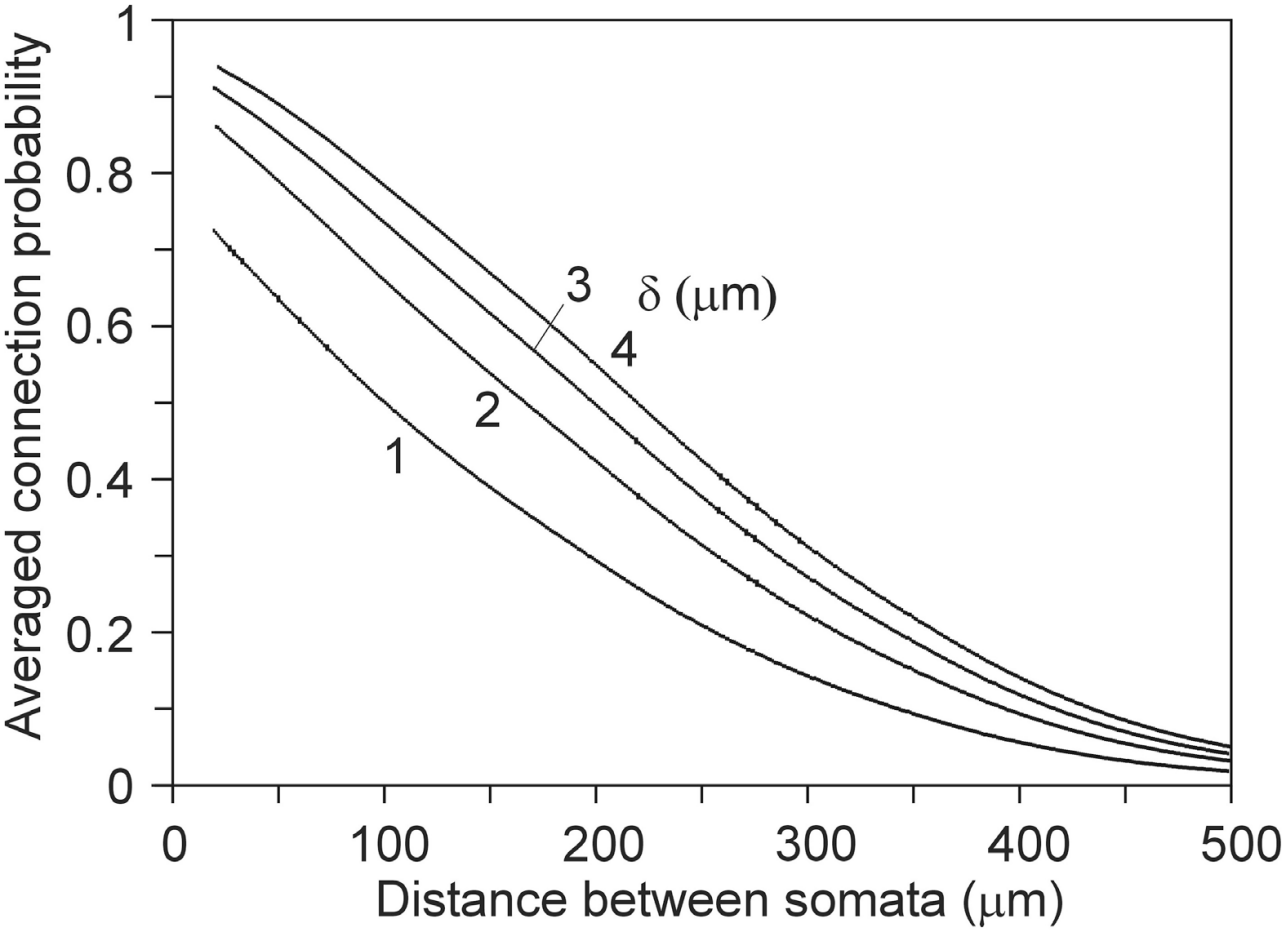
**Network connection probabilities, averaged over all neuron pairs in the network, as a function of their Euclidean intersoma distance**. A number of 2000 somata were uniform randomly distributed in a cylinder with a height of 360 μm and a diameter of 1000 μm.

For deriving the mean connection probability in a network one needs to average over all the different mutual positions of the neuron pairs. This can be done by calculating the distance distributions of all the neuron pairs in the network and convoluting the distributions with the expected connection probabilities, as shown in Figure 7. An example is given in Figure 12 for a network composed of 2000 neurons, all represented by the same population mean density field, obtained from the data set of 50 neurons (see Figure 2). The somata of the 2000 neurons were uniform randomly distributed in a cylindrical space with a height of 360 μm and a diameter of 1000 μm.

#### Estimation of euclidean distances of synapses to their pre- and post-synaptic somata

Density fields can also be used to derive the Euclidean distance distributions of synapses to their pre- and post-synaptic somata. To this end, the probability of finding a synapse is determined in each voxel in the overlap area as well as the voxel's Euclidean distance to pre- and post-synaptic somata. The distance distributions are then constructed by summing the probabilities sorted by their distances. Evidently, pathlength distributions of synapses to their pre-and post-synaptic somata cannot be determined, as the arbor structure is lost in creating the density fields.

**Figure 13:**
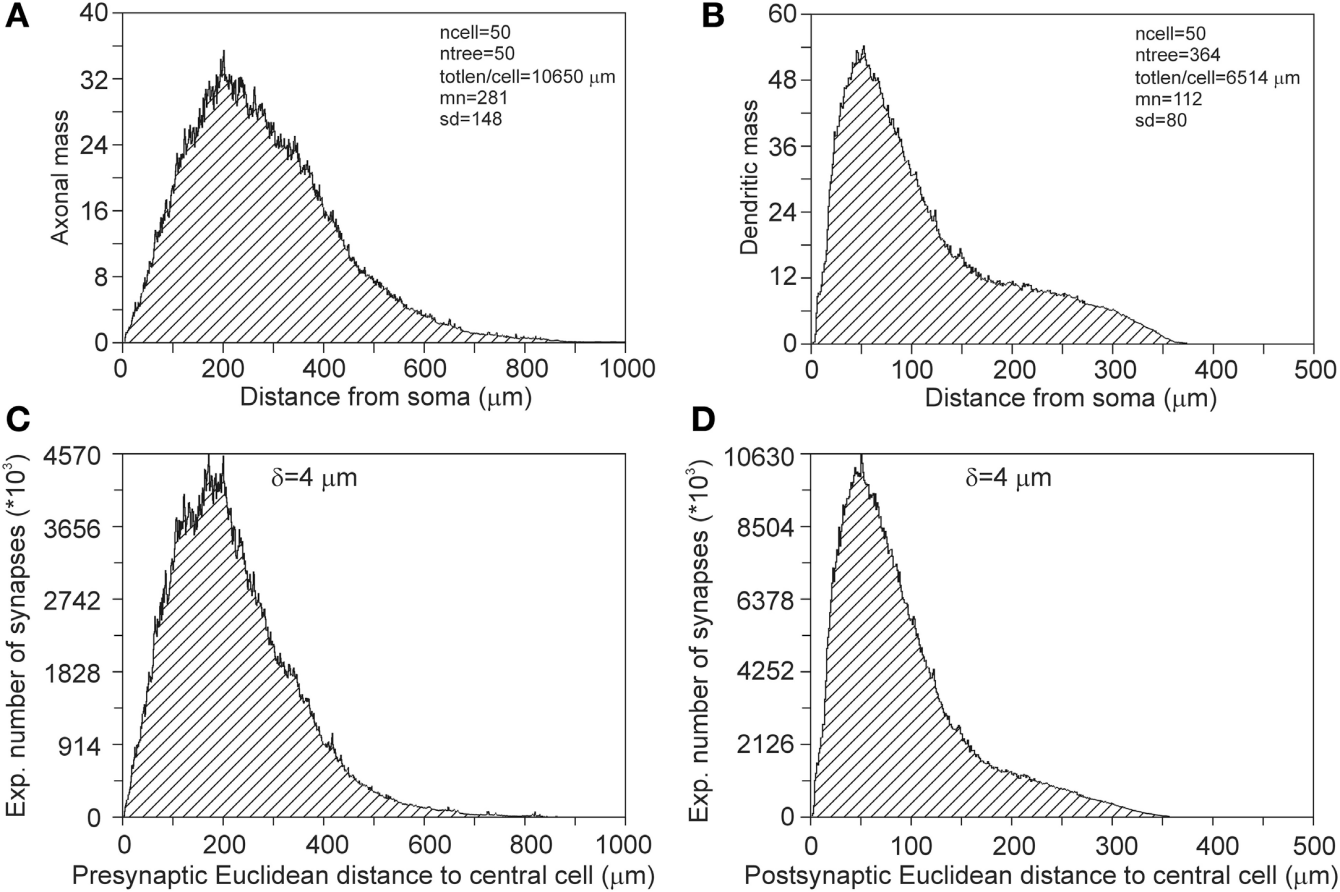
**(A)** Axonal and **(B)** dendritic mass distributions vs. Euclidean distance to somata. The tail in the dendritic mass distributions originates from the apical dendrite and its apical tuft. **(C)** Pre-synaptic and **(D)** post-synaptic Euclidean distance distributions of a centrally located neuron, as calculated from its connections with all other neurons. The total number of 5000 neurons are uniform randomly distributed in a cylinder of height 360 μm and radius 1000 μm. The pre-synaptic distance distribution has a mean(*sd*) value of 216 (120) μ m and the post-synaptic distance distribution a mean(*sd*) of 91(63) μ m.

Synapses can occur only where axons and dendrites overlap in space. These overlap areas are determined by the positions of the somata and the extents of their arbors. When a dendrite overlaps only with remote areas of an axonal field, the possible synaptic locations will have large Euclidean distances to their pre-synaptic somata. Alternatively, when a dendrite overlaps with central areas of an axonal field, possible synaptic locations will have short Euclidean distances to their pre-synaptic somata. When synapses are distributed homogeneously over the axonal and dendritic arborizations, their Euclidean distance distributions reflect the axonal and dendritic mass distributions vs. Euclidean distance to their somata (Figure 13).

To illustrate the effect of spatial boundaries, we calculated the pre- and post-synaptic distances for a centrally located neuron in the cylindrical space of height 360 μm and diameter 2000 μm, with a total number of 5000 neurons (density of 4421 neurons/mm^3^) that are uniform randomly distributed in the cylindrical space (Figures 13C,D). Note that the expected number of synapses between two neurons vs. their intersoma distance follows the patterns as shown in Figure 4. Although the pre-synaptic distribution (Figure 13C) has a rough resemblance with the axonal mass distribution in Figure 13A, it differs from that, with a mean distance of 281 μm for the mass distribution and a mean distance of 216 μm for the pre-synaptic distribution. Also the post-synaptic distribution (Figure 13D) has a rough resemblance with the dendritic mass distribution in Figure 13B, but differs in particular in the tail of the distribution, with a mean distance of 112 μm for the mass distribution and a mean distance of 91 μm for the post-synaptic distribution.

**Figure 14:**
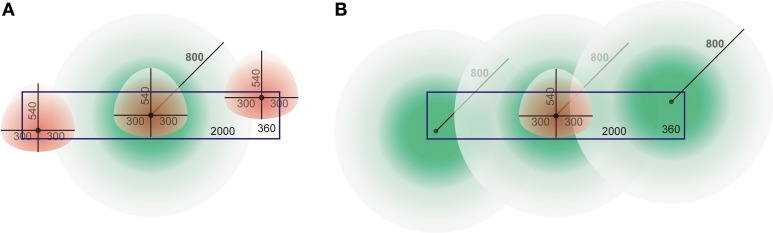
**Cartoon drawing of axonal (green) and dendritic (red) density fields with their somata in a cylindrical space (blue rectangle) of height 360 μm and diameter 2000 μm. (A)** An axonal field with its soma centered, and three dendritic fields at various locations in the space. **(B)** A dendritic field with its soma centered, and three axonal fields at various locations in the space.

These differences can be understood from a cartoon drawing illustrating the dimensions of the dendritic and axonal density fields and the cylindrical space. Figure 14A shows an axonal density field of a centrally located neuron in the cylindrical space and three dendritic density fields of neurons at nearby and remote locations. The figure illustrates that a large part of the axonal field of the central neuron cannot be overlapped by dendritic fields of the other neurons due to their spatial constraints within the cylinder. At low cell densities, the shape of the pre-synaptic distance distribution becomes in a sensitive way dependent on the particular locations of the dendritic density fields (not shown here). This was not so much the case for the post-synaptic distributions. Because of the size of the axonal fields the central dendritic density field will have overlap with many more axonal fields (Figure 14B), even at low cell densities. Because of the spatial constraint, the apical part of the dendritic density field will generally be overlapped by a less dense part of the axonal density fields; this explains why the tail in the post-synaptic distributions differs from that in the dendritic mass distribution.

**Figure 15:**
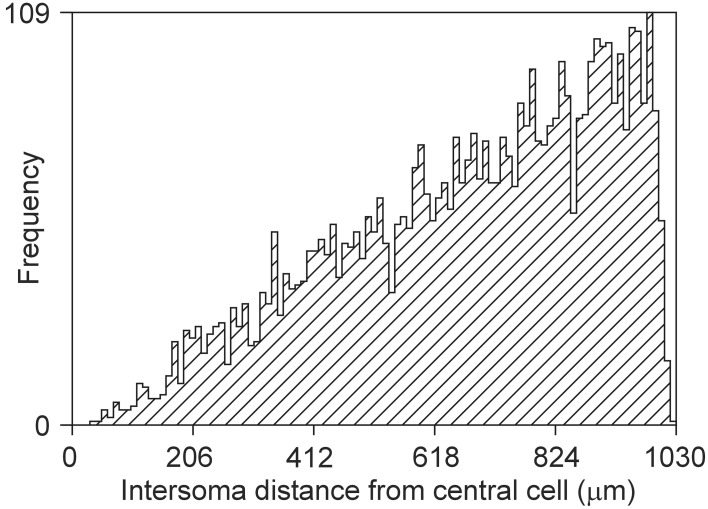
**Frequency distribution of distances between a central soma and 5000 other somata uniform randomly distributed in a cylindrical space of height 360 μm and diameter 2000 μm (density of 4421/ mm^3^)**.

The bounded area of the cylinder also puts constraints on the intersoma distance distribution, as shown in Figure 15. The rather linear pattern differs significantly from a quadratic pattern expected in unconstrained space.

## Discussion

### Rationale and summary

Neuronal density fields are statistical descriptors of the spatial innervation of axonal and dendritic arborizations. They were used in several studies to estimate neuronal connectivity (Uttley, 1955; Liley and Wright, 1994; Kalisman et al., 2003; Stepanyants and Chklovskii, 2005). Recently, we developed a new criterion for determining the location of synaptic contacts in areas innervated by both dendritic and axonal arborizations (van Pelt et al., 2010). In order to apply this criterion to connectivity studies based on density fields, we needed to develop new methodology. A second objective of the present study was to validate the connectivity estimates based on the density field approach with the connectivity data derived from the actual arborizations.

Our recently developed method for finding synaptic locations is based on crossing dendritic and axonal line pieces in combination with a distance criterion (van Pelt et al., 2010). The application of this criterion to density fields required an investigation into the statistical geometry of intersections of lines and voxels (Appendix section A1). First we needed to obtain intersections of randomly oriented lines with cubic voxels, a procedure that turned out to be not trivial. The intuitive procedure of first selecting a uniform random point within the cube through which a uniform oriented line is drawn was incorrect. Essential is that first a uniform random orientation is selected followed by the selection of a uniform random point in space (thus not restricted by the cube) through which the line is drawn. The length distributions of the intersections were highly irregular. Their orientations (in terms of azimuth and elevation angle distributions) were significantly different from those expected for random oriented lines (showing uniform and cosine distributions, respectively). We were not able to trace earlier literature on these topics; thus to our knowledge these findings are new. For sake of completeness, the 2D case for intersections of random lines with a square in a plane has been included in Appendix section A1.

Knowing the mean intersection length makes it possible to relate the density in a voxel to the probability of an intersection. By taking a random “dendritic” intersection in a given voxel and a random “axonal” intersection in another voxel, we were able to apply the crossing/proximity criterion. If both line pieces cross and the crossing distance between the line pieces was within the distance criterion, a synaptic connection was identified. Repeating this procedure many times yielded the probability of a synaptic connection, weighted by the intersection probabilities for this voxel pair. The connectivity of a given “dendritic” voxel could be obtained by pairing it with all “axonal” voxels in its close environment. The total sum for all dendritic voxels in the overlap area of the axonal and dendritic density fields resulted in the expected number of contacts between the “axonal” and “dendritic” neuron.

The summation over all local “axonal voxels” around a dendritic voxel can be simplified if the local axonal densities do not vary much. Then the summation can be replaced by the product of the axonal and dendritic density in the dendritic voxel only, multiplied with a *local environment crossing factor* that integrates the crossing properties of random dendritic line pieces in the dendritic voxel and random axonal line pieces in the local environment. This factor is independent of the density fields, and thus can be obtained once and applied to all voxel pairs. For smooth axonal density fields without strong gradients, this assumption is warranted, but for individual neuron density fields it may not. To test the error made in such conditions, we calculated the expected number of contacts between neurons using their individual density fields with the exact procedure and the approximated one. Both procedures yielded similar results as long as the expected number of contacts was not too small. For very small values, however, for instance in the case of large intersoma distances, the approximation procedure underestimated the number of contacts compared with the exact procedure, down to even 100% (Figure 11). However, averaged over neuron pairs for a range of intersoma distances, the relative difference was less than about 0.2%.

With the approximation expression, the expected number of synaptic contacts between two neurons reduces to a simple summation over all the voxels in the overlap area of the axonal and dendritic density product per voxel, multiplied with *I*_coef_ (which includes the *local environment crossing factor*) (Equation A28, and **Table A1**). The expression derived by Liley and Wright (1994) had a similar structure but with an integral of density products, because of the formulation in continuous space. The coefficient in their expression was equal to $\frac{\pi \varepsilon }{2}$, with ε denoting the distance criterion. This coefficient turned out to be equal to our coefficient *I*_coef_ (at least up to the 3rd decimal, **Table A4**, and see Equation A39). This proves the consistency between the two fully independent and different approaches.

### Estimation of connectivity measures

The calculations were based on a data set of 50 neurons generated with the simulator NETMORPH (Koene et al., 2009). The growth rules were optimized on a data set of rat cortical L2/3 pyramidal cells from the NeuroMorpho.org database. For the calculation of the density fields the neurons were aligned according to their apical dendrites, and axial symmetry was assumed. Although the population mean density fields were far from smooth (particularly in remote areas; see Figure 3), accurate estimates could be obtained for the connectivity measures between neuron pairs at varying locations of their somata.

An important objective of this study was the validation of the density-field based connectivity expectations with the data obtained from the actual arborizations.

#### Validation of the estimation of the number of contacts

As shown in Figure 5, the agreement between both approaches for calculating the number of contacts was extremely good, even within the small standard error in the mean of the actual arborizations (because of averaging over 2450 neuron pairs). This implies that the number of contacts estimated using population mean density fields is a full alternative to the averaging over the number of contacts between the actual neuronal arborizations.

#### Estimation of the connection probability

An attempt was made to estimate the connection probability from the density fields. A basic assumption in the approach used was that synaptic contacts are independently distributed in 3D space. The incorrect outcomes made clear that this assumption was not valid. Actually, it emphasizes the correlative structure in the spatial distribution of synapses, which may not be surprising as synapses are distributed along neuronal arborizations. These correlative structures are not preserved in the population mean density fields, making density fields not suitable for predicting connection probabilities. Alternatively, we estimated the connection probabilities from the correct expected number of contacts by using empirical mapping functions, which produced outcomes that agreed very well with the validation data.

#### Estimation of the number of contacts per connection

Because this connectivity measure is calculated as the ratio of the expected number of contacts and the connection probability, it cannot be estimated from the density fields either. Alternatively, we estimated the number of contacts per connected neuron pair from the correct expected number of contacts by using empirical mapping functions, which produced outcomes that agreed very well with the validation data.

#### Empirical mapping functions

The empirical mapping functions for both the connection probability and the number of contacts per connection were dependent on the distance criterion for synaptic contacts. Whether these mapping functions are also dependent on the morphology of the cell types is still unknown. If not, the mapping functions could have a general validity. Investigation of this question was considered to be outside the scope of this paper.

#### Distinction between basal and apical dendrites of pyramidal cells

In the calculation of the dendritic density fields, no distinction was made between basal and apical dendrites. When such a distinction is made, the connectivity measures can be estimated for basal and apical dendritic connectivity separately.

### Comparison of present findings with other connectivity studies

#### Number of contacts between two neurons

Hellwig (2000) estimated computationally the number of contacts between eight experimentally reconstructed rat cortical L2/3 pyramidal neurons by placing them at several distances from each other. For two groups of four neurons each, the number of contacts as a function of the cell separation was determined using a distance criterion of 1 μm. Our results (Figure 5) compare well with the two regression curves of Hellwig. The difference between the two regression curves of Hellwig illustrates the effect of small sample sizes (four) when the number of contacts between individual neuron pairs may vary as strongly as shown in Figure 10 (see also McAssey et al., in revision).

#### Connection probabilities in neuronal networks

In neuronal networks neurons take different positions. To derive connectivity estimates for the whole network, one needs to average over all the different relative positions of the neuron pairs. This can be done by calculating the distance distributions of all the neuron pairs and convoluting the distributions with the expected connectivity data (see Figures 5, 7, 9). An example of this procedure for calculating the averaged connection probability is given in Figure 12. For a distance criterion of 1 μm, the connection probability shows a monotone decreasing pattern, from a value of about 0.7 at very short intersoma distances down to about 0.04 at an intersoma distance of 500 μm. For larger distance criteria the connection probabilities slightly increase, while the intersoma distance dependency becomes more linear. Experimental data on connection probabilities of rat layer 2/3 rat pyramidal neurons have been collected by Holmgren et al. (2003). In paired electrophysiological recordings, they found connection probabilities of about 0.09 at intersoma distances of 0–25 μm, decreasing down to about 0.01 at intersoma distances of 100–200 μm. Using multipatch experiments on a large set of thick-tufted layer 5 pyramidal neurons in rat cortical somatosensory slices, Perin et al. (2011) estimated the mean (functional) connection probability as a function of intersoma distance. The connection probabilities for this type of neuron showed a similar dependence on distance but with values about a factor of 3–4 lower than our outcomes.

In general, our estimates are substantially higher than the experimental estimates. Several notes need to be made. Our estimates are based solely on geometrical considerations and mark only possible candidate synaptic locations. Whether at these locations actual synapses are present and whether they are functional and measurable in electrophysiological experiments are open questions. It is notoriously hard to collect experimentally reliable estimates of connectivities in neuronal networks, an effort that is hampered by issues such as cutting effects in slices, unbiased sampling of patched neurons, and measuring resolutions. The computational predictions strongly depend on the chosen distance criterion for synapse formation and, although a criterion of about 4 μm seems plausible in view of the local geometry, it still has to be validated. A larger uncertainty is the probability that a candidate synapse location really represents a functional synapse. With a computational estimate of about 0.9 for the connection probability at very short distances and an experimental estimate of about 0.09 (Holmgren et al., 2003), there is still a factor of 10 difference to be explained.

An interesting finding from the present study is that the expected number of contacts was highest when the pre-synaptic neuron was placed about 50 μm above the post-synaptic neuron (Figure 4). Kalisman et al. (2003) reported a similar observation (with a maximal number of contacts at a displacement of 100 μm for layer 5 pyramidal cells).

#### Pre- and post-synaptic euclidean distance distributions

The probability of having a synapse at a particular location in space directly depends on the local values of the axonal and dendritic densities. The distribution of synapses on the axonal and dendritic arborizations is thus determined by the overlap profile of the density fields, which depends on the locations of the somata. An example is given for a number of neurons with their somata uniform randomly distributed in a cylindrical space (Figure 13). In the case of uniform density fields and unrestricted space, one would expect pre- and post-synaptic distances to be equal to the radial mass distributions of the axons and dendrites. The comparison thus shows the effect of inhomogeneous density fields and restricted space on the spatial distribution of synapses.

Feldmeyer et al. (2002) studied connectivity between layer 4 spiny neurons and the dendrites of layer 2/3 pyramidal cells in the rat barrel cortex by means of paired recording and reconstruction techniques. The distribution of the post-synaptic Euclidean distances of synapses on the pyramidal dendrites turned out to correspond quite well with our predictions, although the limited number of their observations (59 synapses in 13 neuron pairs) prevented a detailed shape comparison. Data on pre-synaptic Euclidean distance distributions appears to be absent in the literature, probably because of the experimental challenges involved in reconstructing full axonal arbors of neurons that project to a given target neuron.

### Experimental challenges in measuring network connectivity

Helmstaedter (2013) recently evaluated the state-of-the art of experimental techniques for resolving the connectivity matrix in neuronal circuits (connectomics). As structures involved in connections (axonal diameters, spine necks) have minimal dimensions of less than about 50 nm, the minimal required imaging resolution must be less than about 30 nm. Present electron microscopy techniques meet these resolution requirements but are limited in the volume that they can image. While these reconstructions are time consuming, the time needed for segmentation and determining the wiring exceeds these imaging times by factors. Therefore, computational approaches may provide valuable alternative approaches for studying connectivity at a cellular level in neuronal networks.

### Future challenges

#### Density fields calculated from experimentally reconstructed neurons

The present study was based on a set of neuronal morphologies produced by the simulator NETMORPH (Koene et al., 2009), but could equally well have been based on a set of experimentally reconstructed neurons. A set of simulated rather than experimentally reconstructed neurons was chosen because it puts no restriction on the number of neurons and because simulated neurons do not suffer from incompleteness caused by tissue sectioning, a problem that affects many sets of experimentally reconstructed neurons.

If a sufficiently large set of experimentally fully reconstructed neurons became available, the axonal and dendritic density fields derived from these neurons would provide powerful statistical representations of their spatial innervation patterns. These density fields can replace actual arborizations when one wants to build networks of these neurons. The limited availability of actual neuronal reconstructions is then no longer restricting the size of the network. The connectivities emerging in such networks can then be reliably estimated from the overlap of the neurons' density fields, as has been shown in this study. For building cortical networks, one needs density fields of a variety of neuron types. These neuron-specific density field templates are not yet available, and constructing them would be an interesting challenge for the future.

#### Variability in neuronal morphologies and density fields

Neurons vary substantially in their morphologies. Density fields based on different data sets from the same neuron population will also show variations, which inevitably propagate to variations in the estimated connectivity values. This issue has recently been addressed by McAssey et al. (in revision). They show how the variation in the estimated number of contacts between two neurons decreases with increasing size of the data set used for calculating the density fields. They advocate the use of neuronal simulators, because simulators enable the generation of any desired number of morphologies so that the density fields can be estimated with any desired level of statistical stability. Essential is that the simulated neurons are realistic in all relevant aspects of their morphology.

#### Density field completion of sectioned incomplete neurons

Many neuronal reconstructions for a variety of cell types and species that are made available through the NeuroMorpho.Org data base (Ascoli, 2006) are, unfortunately, incomplete and not directly suitable for constructing density fields. However, when parts of the density fields within the spatial constraint of a section can be reliably estimated from incomplete neuronal reconstructions, it should be possible to make the density field complete provided axial or spherical symmetry can be assumed.

#### Density fields of neuronal populations at various developmental stages

During neuronal development, axonal and dendritic arbors increase their spatial innervation area by neurite elongation and branching. Connectivity studies on developing networks critically rely on the availability of reconstructed neurons at different developmental stages, but such morphological time series are unfortunately scarce. Density fields of outgrowing neurons will also change with developmental stage and presumably according to a particular growth pattern. If density fields can be determined for a number of developmental stages, such growth patterns could possibly be described in terms of a density field growth function. These density field growth functions could then, for example, be used for (i) interpolating or extrapolating to developmental stages for which experimental data is not available, and (ii) studying connectivity in developing neuronal networks.

## Conclusion

Determining the connectivity between neurons requires knowledge about their innervation of space. Neurons can be represented by their actual arborizations, but also by their density fields. In this paper, we have shown that the number of contacts between neurons estimated from their population mean density fields is fully consistent with the number of contacts calculated from their actual arborizations. However, the connection probability and the number of contacts per connection cannot be reliably estimated from the density fields. Alternatively, they can be estimated from the expected number of contacts by using empirical mapping functions. The population mean density fields are powerful representations of the mean axonal and dendritic spatial innervation patterns of a given cell type. These density fields can be used in neuronal network studies to obtain statistical connectivity estimates by representing each neuron by the population mean density field of its cell type. The large variation between individual neurons is then already expressed in the density field itself.

### Conflict of interest statement

The authors declare that the research was conducted in the absence of any commercial or financial relationships that could be construed as a potential conflict of interest.

## Supplementary material

The Appendix of this article is supplied as Supplemental Data (Data Sheet.pdf) and can be found online at: http://www.frontiersin.org/journal/10.3389/fncom.2013.00160/abstract

## References

[B1] Ascoli G. A. (2006). Mobilizing the base of neuroscience data: the case of neuronal morphologies. Nat. Rev. Neurosci. 7, 318–324 10.1038/nrn1885 16552417

[B2] Binzegger T. Douglas R. J. Martin K. A. C. (2004). A quantitative map of the circuit of cat primary visual cortex. J. Neurosci. 24, 8441–8453 10.1523/JNEUROSCI.1400-04.2004 15456817 PMC6729898

[B3] Cuntz H. (2012). The dendritic density field of a cortical pyramidal cell. Front. Neuroanat. 6:2 10.3389/fnana.2012.00002 22347169 PMC3269636

[B4] Feldmeyer D. Lübke J. Silver R. A. Sakmann B. (2002). Synaptic connections between layer 4 spiny neurone-layer 2/3 pyramidal cell pairs in juvenile rat barrel cortex: physiology and anatomy of interlaminar signalling within a cortical column. J. Physiol. 538, 803–822 10.1113/jphysiol.2001.012959 11826166 PMC2290091

[B5] Glaser E. M. McMullen N. T. (1984). The fan-in projection method for analyzing dendrite and axon systems. J. Neurosci. Methods 12, 37–42 10.1016/0165-0270(84)90045-1 6513589

[B6] Hellwig B. (2000). A quantitative analysis of the local connectivity between pyramidal neurons in layers 2/3 of the rat visual cortex. Biol. Cybern. 82, 111–121 10.1007/PL00007964 10664098

[B7] Helmstaedter M. (2013). Cellular-resolution connectomics: challenges of dense neural circuit reconstruction. Nature Methods 10, 501–507 10.1038/nmeth.2476 23722209

[B8] Holmgren C. Harkany T. Svennenfors B. Zilberter Y. (2003). Pyramidal cell communication within local networks in layer 2/3 of rat neocortex. J. Physiol. 551, 139–153 10.1113/jphysiol.2003.044784 12813147 PMC2343144

[B9] Kalisman N. Silberberg G. Markram H. (2003). Deriving physical connectivity from neuronal morphology. Biol. Cybern. 88, 210–218 10.1007/s00422-002-0377-3 12647228

[B10] Koene R. A. Tijms B. Van Hees P. Postma F. De Ridder S. Ramakers G. (2009). NETMORPH: A framework for the stochastic generation of large scale neuronal networks with realistic neuron morphologies. Neuroinformatics 7, 195–210 10.1007/s12021-009-9052-3 19672726

[B11] Liley D. T. J. Wright J. J. (1994). Intracortical connectivity of pyramidal and stallate cells: estimates of synaptic densities and coupling symmetry. Network 5, 175–1989 10.1088/0954-898X/5/2/004

[B12] Perin R. Berger T. K. Markram H. (2011). A synaptic organizing principle for cortical neuronal groups. Proc. Natl. Acad. Sci. U.S.A. 108, 5419–5424 10.1073/pnas.1016051108 21383177 PMC3069183

[B13] Peters A. (1979). Thalamic input to the cerebral cortex. Trends Neurosci. 2, 1183–1185 10.1016/0166-2236(79)90074-2

[B14] Shepherd G. M. Svoboda K. (2005). Laminar and columnar organization of ascending excitatory projections to layer 2/3 pyramidal neurons in rat barrel cortex. J. Neurosci. 25, 5670–5679 10.1523/JNEUROSCI.1173-05.2005 15958733 PMC6724876

[B16] Stepanyants A. Chklovskii D. B. (2005). Neurogeometry and potential synaptic connectivity. Trends Neurosci. 28, 387–394 10.1016/j.tins.2005.05.006 15935485

[B17] Uttley A. M. (1955). The probability of neural connexions. Proc. R. Soc. Lond. B Biol. Sci. 144, 229–240 10.1098/rspb.1955.0054 13266808

[B18] van Pelt J. Carnell A. De Ridder S. Mansvelder H. D. van Ooyen A. (2010). An algorithm for finding candidate synaptic sites in computer generated networks of neurons with realistic morphologies. Front. Comput. Neurosci. 4:148 10.3389/fncom.2010.00148 21160548 PMC3001749

