# Supplementary material for: Estimating neuronal connectivity from axonal and dendritic density fields
Source: Front Comput Neurosci. 2013 Nov 25;7:160. doi: 10.3389/fncom.2013.00160 (PMC3839411; doi:10.3389/fncom.2013.00160)
Supplement: Supplementary file 1 [file DataSheet1.PDF]

Estimating neuronal connectivity from axonal and dendritic density fields

Jaap van Pelt and Arjen van Ooyen

APPENDIX

This APPENDIX is an integral part of the article that can be found online at: http://www.frontiersin.org/journal/10.3389/fncom.2013.00160/abstract

	Page
A1. Uniform random lines in 2D and 3D and their intersections with squares and cubes	2
A1.1. Uniform random line intersections of a square in 2D	2
A1.2. Uniform random line intersections of a cube in 3D	2
A1.3. Other procedures for generating random (but biased) line intersections within a square or cube	3
A2. Density fields	4
A2.1. Voxel hit probabilities and field densities	4
A3. Crossing of random lines	5
A3.1. Crossing of random line intersections within a single cube	5
A3.2. Crossing of random line intersections in different cubes	6
A4. Conditional crossing probability	6
A4.1. Conditional crossing probability of random line pieces in a single cube of arbitrary size	6
A4.2. Conditional crossing probability of random line pieces in voxels at a given distance	7
A5. Overlapping density fields	7
A5.1. Expected number of crossing line pieces in an overlap area of two density fields.	7
A5.1.1. Overlap of individual neuron density fields	8
A5.2. Approximation – uniformity in axonal densities in the local environment of dendritic voxels	9
A5.2.1. Local environment crossing factor $\mathrm{f}^{\mathrm{env}}(\mathrm{s},\delta)$ - sum of unweighted conditional crossing probabilities over all voxels in the local environment of a given voxel *v*	10
A5.2.2. Local environment crossing factor $\mathrm{f}^{\mathrm{env}}(\mathrm{s},\delta)$ - local environment as a single cube of size equal to a block of voxels	10
A5.2.3. Unconditional unweighted crossing probabilities of random line pieces in centered cubes of arbitrary size	10
A5.2.4. Conditional weighted crossing probabilities of random line pieces in a central voxel and a centered cube	12
A5.2.5. Overlap sum coefficient I_{coef} and closed expression $E\{n_{D,A}^{cross}\|\delta\}$	13
A5.3. Crossing distances between crossing line piece pairs in centered cubes of different size	13
A6. Invariance of the scale of the grid for the calculation of crossing probabilities	14
A7. Estimating the connection probability and the expected number of contacts per connected neuron pair from the expected number of synaptic connections	15

A1. UNIFORM RANDOM LINES IN 2D AND 3D AND THEIR INTERSECTIONS WITH SQUARES AND CUBES

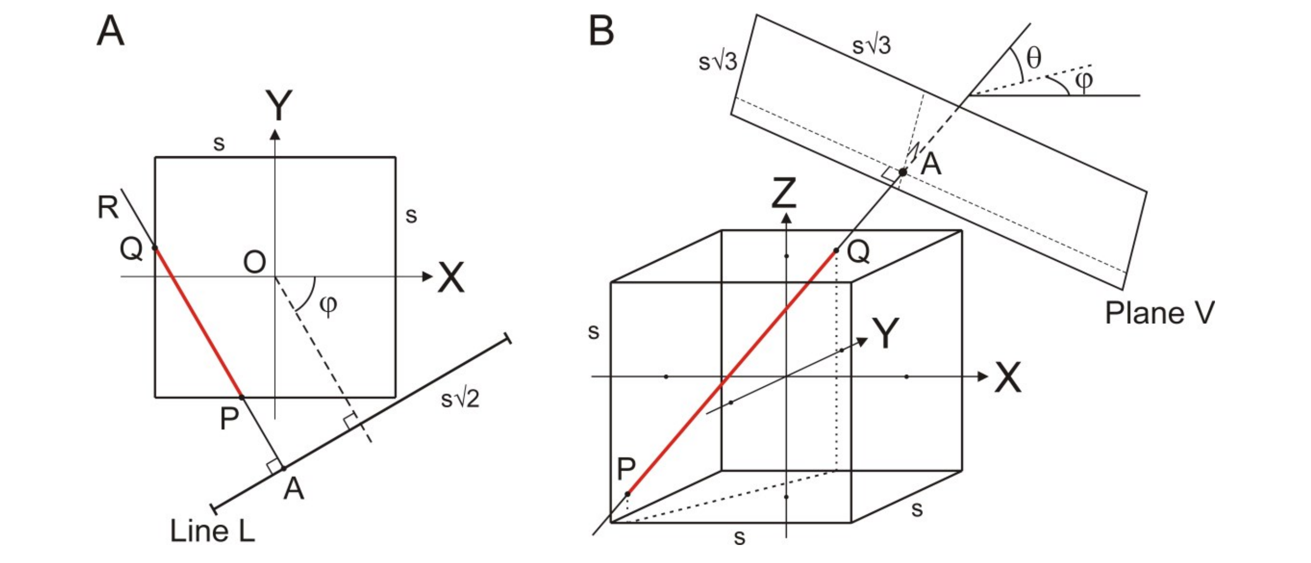

FIGURE A1 | Uniform random line intersections in (A) a square and in (B) a cube.

A1.1. UNIFORM RANDOM LINE INTERSECTIONS OF A SQUARE IN 2D

A uniform random line in 2D space is a line with a uniform random orientation drawn from a random point in the 2D space. A uniform random orientation is obtained by taking a uniform random angle with respect to the X-axis in the interval [-90,90] degrees via

$$\varphi_R = -90 + 180 \times rand \qquad \text{(A1)}$$

with *rand* indicating a uniform random number in [0,1). The selection of a uniform random point in 2D space through which the line is drawn can be simplified by taking a uniform random point on a 1D infinite line perpendicular to the random orientation ('look-up line'). When it concerns the selection of a random line through a square, the infinite 'look-up' line can be reduced to a finite 'look-up' line piece (L) of such a size that it covers the full square, as seen from any orientation of the random line (Fig. A1A). Then it suffices to select a uniform random point (A) on this finite 'look-up' line piece through which the random line is drawn. It is crucial that the 'look-up' line pieces are of the same size for any orientation of the random line, to guarantee that the square is seen by line beams with the same line density for any orientation. For a square of size s, the 'loop-up' line should have a length of at least $s\sqrt{2}$ (the length of the diagonal in the square). Lines with the given random orientation and drawn from random points on the 'look-up' line piece may or may not hit the square, depending on the orientation of the square with respect to the line. The probability of hitting the square is proportional to the length of the projection of the square onto the 'loop-up' line piece. This fact guarantees that the square is probed with constant density line beams for any orientation of the beam.

The intersection of a random line with a square is indicated by the points P and Q in Fig. A1A. The length distributions of these intersections are displayed in the graphs of Fig. A2. The mean (sd) intersection length is equal to

$$\overline{PQ_{square}} = 0.7856 \times s\ (0.3555 \times s)\ . \qquad \text{(A2)}$$

The intersection length distribution for a square (Fig. A2A) has a complex shape, and is composed of two components. It has one component for the intersections of opposite sides which have a length range of $[s, s\sqrt{2}]$ (Fig. A2B) and one component for the intersections of two neighboring sides which have a length range of $[0, s\sqrt{2}]$ (Fig. A2C). A particular property of the angle distribution of the intersecting line pieces is that it is not uniform but has maxima at angles of -45 and 45 degrees (Fig. A2D). This is caused by the fact that lines with these orientations see the square from a diagonal point of view, such that the square captures most of the random lines (a square is not a circular object). A typical set of intersections of uniform random lines with a square is shown in Fig.A2E.

A1.2. UNIFORM RANDOM LINE INTERSECTIONS OF A CUBE IN 3D

The generation of uniform random lines in 3D follows the same principle as in 2D. A random line in 3D space is a line with a uniform random orientation drawn from a random point in 3D space. A uniform random orientation is obtained by taking a uniform random azimuth angle with respect to the X-axis in the interval [-90,90] degrees via Eq. (A1) and a sine-weighted random elevation angle θ via

$$\theta_R = \arcsin(2 \times rand - 1)\ . \qquad \text{(A3)}$$

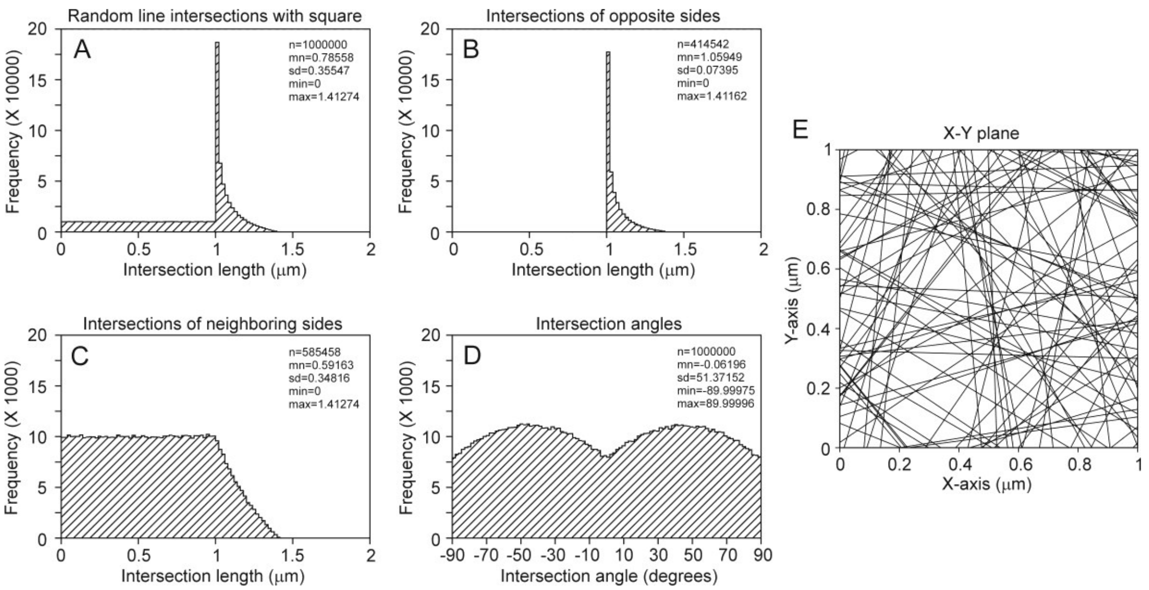

FIGURE A2 | Intersections of uniform random lines with a square of size s_s=1 μm. Shown are **(A)** the length distribution of the line intersections, **(B)** the length distribution of line intersections of opposite sides of the square, **(C)** the length distribution of line intersections of neighboring sides of the square, **(D)** the angle distribution of the intersections, as well as **(E)** a typical set of random line intersections.

The selection of a uniform random point in 3D space through which the line is drawn can be simplified by taking a uniform random point in a 2D infinite plane perpendicular to the random orientation ('look-up plane'). When it concerns the selection of a random line through a cube, the infinite 'look-up' plane can be reduced to a finite 'look-up' plane (V) of such a size that it covers the full cube, as seen from any orientation of the random line (Fig. A1B). Then it suffices to select a uniform random point (A) on this finite 'look-up' plane through which the random line is drawn. It is crucial that 'look-up' planes of the same size are taken for any orientation of the random line, to guarantee that the cube is seen by line beams with the same line density for any orientation. A cube of size s is, for any angle, fully covered by a square of size $s\sqrt{3}$, the length of the diagonal in the cube. Lines with the given random orientation and drawn from random points on the 'look-up' square may or may not hit the cube. The number of hits depends on the orientation of the cube with respect to the line, because the number of hits is proportional to the projection area of the cube onto the 'look-up' plane. This fact guarantees that the cube is probed with constant density line beams for any orientation of the lines.

The intersection of a uniform random line with a cube (in the following denoted by a random line piece in a cube) is indicated by the intersecting points P and Q in Fig. A1B. The length distribution of the intersections of the cube by uniform random lines (Fig. A3A) has a mean (sd) of

$$\overline{PQ_{cube}} = 0.66653 \times s \ (0.39156 \times s) \ . \qquad \text{(A4)}$$

This distribution has also a complex shape with two components, one of lines intersecting opposite cube side planes (Fig. A3B), and one of random lines intersecting neighboring cube side planes (Fig. A3C). Intersections between neighboring side planes can be as short as zero, and as long as the cube diagonal, thus with range $[0, s\sqrt{3}]$. Intersections of opposite side planes cannot be shorter than the size s of the cube, but can also be as long as the cube diagonal, thus with range $[s, s\sqrt{3}]$. The azimuth angle distribution of the intersecting line pieces (Fig. A3E) does not show a uniform random pattern, but two peaks at the angles of -45 and of 45 degrees. A similar argument to that in the 2D case can be applied here, in that uniform random lines with these orientations see the cube with the largest area and thus with the highest hit probability. The elevation angle distribution (Fig. A3F) deviates from the sine pattern (expected for uniform random line pieces) by having two maxima. They are located at smaller angles than the elevation angles of +35.3 and - 35.3 degrees of the diagonals; this is probably a convolution effect of the original sine shape with the orientation-dependent intersection pattern. A typical set of intersections of uniform random lines wth a cube is shown in Fig. A3G.

A.1.3. OTHER PROCEDURES FOR GENERATING RANDOM (BUT BIASED) LINE INTERSECTIONS WITHIN A SQUARE OR CUBE

Three other procedures were initially explored but with biased results. The first one was to select at random two planes of a cube, and then select a random point on each plane. The connection is then a random line. However, the angle distribution of these lines is biased because line pieces connecting two points in neighboring planes have an angle distribution influenced by the orthogonal orientation of the side planes in the cube.

The second procedure was to select two uniform random points in the cube and then to draw a line between both points. These line pieces, however, have also a biased angle

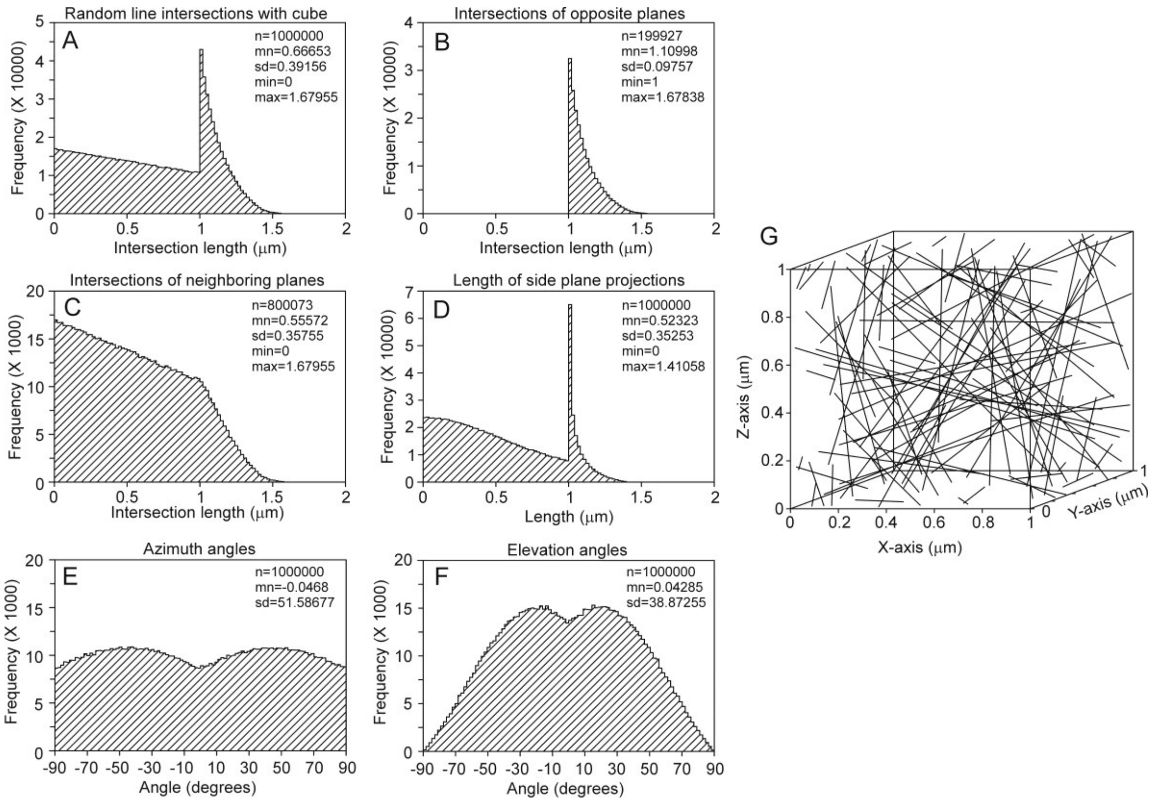

FIGURE A3 | Intersections of uniform random lines with a cube of size s_c=1 μm. Shown are the (**A**) length distribution of random line intersections, (**B**) length distribution of random lines intersecting opposite side planes of the cube, (**C**) length distribution of random lines intersecting neighboring side planes of the cube, (**D**) length distribution of the projection of random line intersections on an arbitrary side plane of the cube, (**E**) azimuth angle distribution of random line intersections, (**F**) Elevation angle distribution of random line intersections, as well as (**G**) 100 intersections of uniform random lines with a cube.

distribution beause in corner areas of the cube the majority of the line pieces originate from points from the main volume of the cube and hence have a clear orientation bias.

The third procedure was to select a uniform random point in the cube and then to draw a line with a uniform random orientation through that point. This procedure results in uniform random azimuth angle distributions and cosine shaped elevation angle distributions. However, these line pieces do also not agree with the characteristics of uniform random lines in space. When applied to the internal sphere of the cube, such a procedure would indeed produce uniform random line intersections. However, when random points are selected in the corner areas of the cube outside the internal sphere, random orientations may result in intersections that do or do not hit the internal sphere. The intersections that also hit the sphere would result in an orientation bias in the intersecting line densities.

A2. DENSITY FIELDS

In a discretized space with voxel size s_v and filled with uniform random lines, each voxel may be hit by these lines resulting in an intersection of a certain length. When a given voxel is hit by multiple lines the total length of its intersections then correlates with the number of hits. The mean length of the intersections in the given voxel is equal to $0.66653 \times s_v$ (Eq. A4), and in a statistical sense the expected total length of the intersections L_v^{tot} can be related to the expected number of hits $E\{n_v^{hit}\}$ as

$$L_v^{tot} = 0.66653 \times s_v \times E\{n_v^{hit}\} = C \times s_v \times E\{n_v^{hit}\} \quad \text{(A5)}$$

with $C = 0.66653$. When the expected number of hits is much smaller than one, we may replace the expectation of the number of intersections by the intersection (hit) probability p_v^{hit} itself (note that under this condition the probability of more than one intersection becomes negligibly small):

$$p_v^{hit} \cong L_v^{tot} / (C \times s_v) \; . \quad \text{(A6)}$$

These expressions are also applicable when the local density of random lines is not uniform. In that case the hit probability p_v^{hit} may differ from voxel to voxel.

A2.1. VOXEL HIT PROBABILITIES AND FIELD DENSITIES

The density field of an arborization reflects the distribution of its 'mass' over the unit grid in 3D space, and the local density ρ_v in each unit voxel denotes the expected amount of 'mass' m_v in that voxel. If mass is expressed in the length of the branches of the arborization, then the density in each unit voxel denotes the expected length L_v^{tot} of the branches in that unit voxel. A voxel of size s_v then contains a total branch

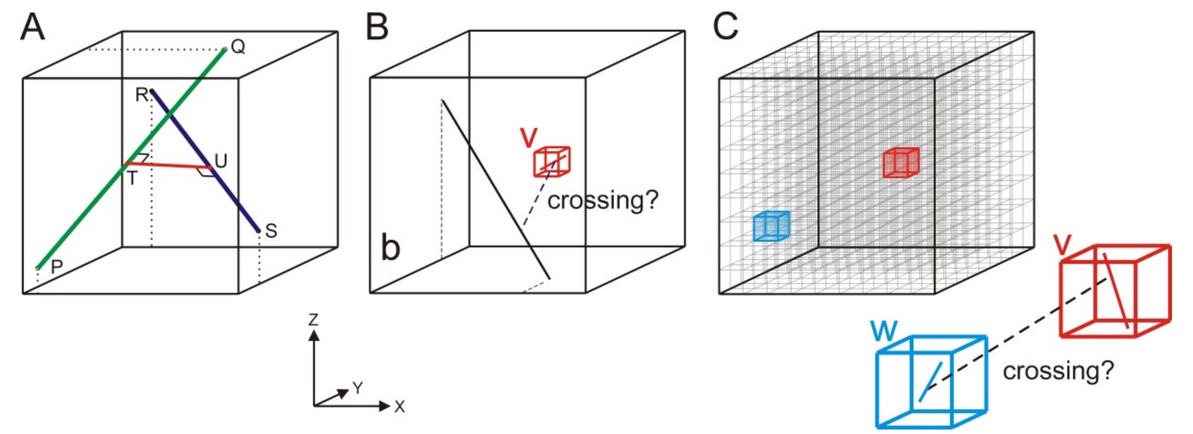

FIGURE A4 | Crossing line pieces in voxels and cubes. Shown are (**A**) two crossing random line pieces PQ and RS in a cube with their crossing distance TU; (**B**) a random line piece in a cube and a random line piece in a voxel; and (**C**) two voxels in a cube within which each a random line.

length L_v^{tot} equal to the local density times the volume of the voxel

$$L_v^{tot} = \rho_v \times s_v^3 \ . \quad \text{(A7)}$$

Conversely, a given total length of branches (line pieces) L_v^{tot} in a voxel of size s_v represents a density of

$$\rho_v = L_v^{tot}/s_v^3 \ . \quad \text{(A8)}$$

Combining Eqs. (A5) and (A7) yields the expected number of intersections of the voxel by the line field:

$$E\{n_v^{hit}\} = L_v^{tot}/C \times s_v = \rho_v \times s_v^2/C \ . \quad \text{(A9)}$$

If this expectation is much smaller than one, we obtain the probability for a voxel to be hit by a branch of the arborization as expressed in terms of the densities:

$$p_v^{hit} \cong \rho_v \ \times s_v^2/C \ . \quad \text{(A10)}$$

A3. CROSSING OF RANDOM LINES

In a recent study, we developed an algorithm for finding synaptic locations in an area innervated by both axonal and dendritic arbors (Van Pelt et al., 2010). The algorithm is based on finding pairs of axonal and dendritic line pieces that cross with a crossing distance smaller than or equal to a given criterion distance δ. In this Appendix, we study the crossing properties of random line pieces in the same or in different voxels.

A3.1. CROSSING OF RANDOM LINE INTERSECTIONS WITHIN A SINGLE CUBE

The crossing probability of two random line pieces in a cube is obtained by generating a large number of pairs of random line pieces (n=1000000) according to the procedure described in section **A1.2.** and by determining for each pair whether they are crossing (Fig. A4A). The crossing probability is equal to the ratio of the number of crossing line piece pairs and the total number of line piece pairs. In the case of crossing line pieces, the length of the orthogonal connection (crossing distance) is determined according to the algorithm described in Van Pelt et al. (2010). The frequency distribution of crossing distances within a single cube of size $s=1$ (Fig. A5) has a mean of 0.334 and a standard deviation of 0.256. The crossing distances scale linearly with the size s of the cube, in such a way that mean $d_{mn}^{cross}(s)$ and standard deviation $d_{sd}^{cross}(s)$ of the crossing distance distribution become

$$d_{mn}^{cross}(s) = 0.334 \times s \ \text{ and } \ d_{sd}^{cross}(s) \ = 0.256 \times s \ . \quad \text{(A11)}$$

The crossing probability of two random line pieces in a cube was found to be equal to

$$p^{cross} = 0.3133 \ , \quad \text{(A12)}$$

which is independent of the size of the cube. Note that for the geometry of (crossing) line pieces in a cube the size of the cube is not relevant.

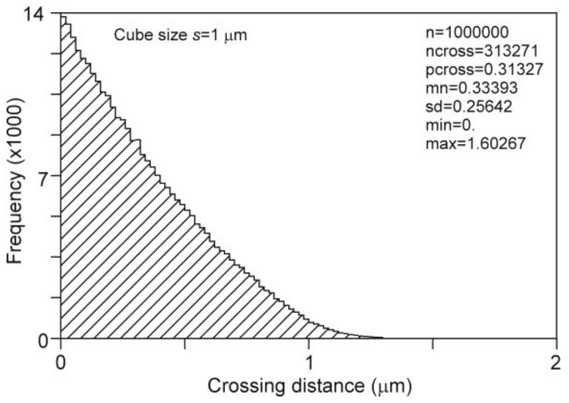

FIGURE A5 | Frequency distribution of the crossing distances between two crossing random line pieces within a voxel of size *s*=1.

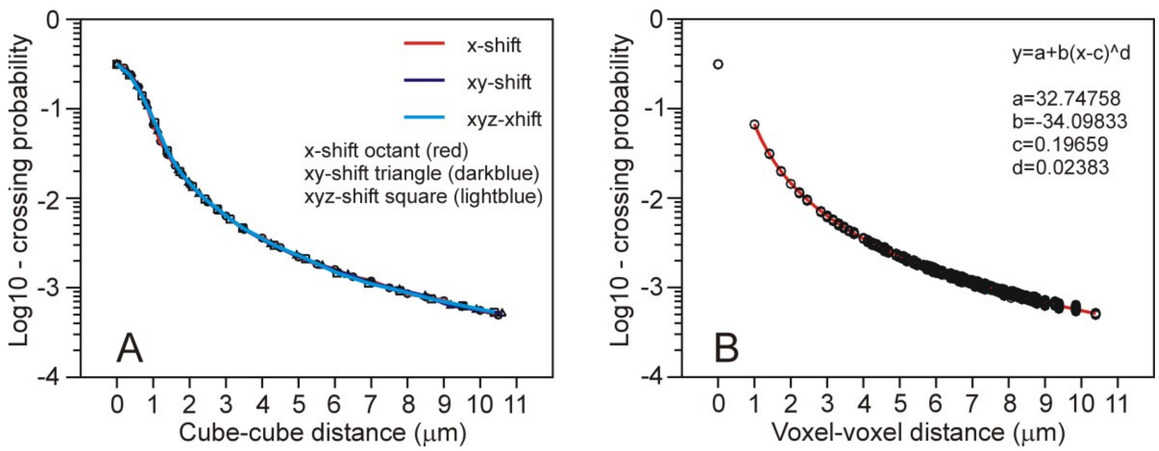

FIGURE A6 | Crossing probabilities of random line pieces in two different unit cubes, plotted versus the distance between both cubes. **(A)** The cubes are stepwise (0.2 μm) shifted in the x-direction (red curve), the xy-diagonal direction (dark blue curve, and the xyz-diagonal direction (light blue curve). (**B**) The random line pieces are taken in the central voxel and in each of the 13^3 =2197 voxels in a block of size *s*=13 μm. The continuous red line is a best fit with a power function, for distances larger than or equal to 1 μm.

A3.2. CROSSING OF RANDOM LINE PIECES IN DIFFERENT CUBES

In a similar manner we can obtain the probability that random line pieces in two different cubes cross (Fig. A4C). The procedure was first to get two cubes v and w of size s with their corners at (x_v, y_v, z_v) and (x_w, y_w, z_w), respectively. The distance $d_{v,w}$ between the cubes is given by

$$d_{v,w} = \sqrt{(x_v - x_w)^2 + (y_v - y_w)^2 + (z_v - z_w)^2}\ . \qquad \text{(A13)}$$

Second, a random line piece was obtained in both cubes, and third it was determined whether the two line pieces were crossing. The crossing distance was obtained according to the algorithm described in Van Pelt et al. (2010). The crossing probabilities, shown in Fig. A6A, are obtained for unit cubes (i.e. with size s=1 μm). The crossing probabilities appear to decrease strongly with the distance between the cubes. The figure contains three curves, calculated for stepwise shifts of 0.2 μm of the cubes in the x-direction (red curve), the xy-diagonal direction (dark blue curve) and the xyz-diagonal direction (light blue curve). The precise overlap between the curves indicates that the crossing probability of random lines pieces in a cube pair is dependent on their Euclidean distance but independent of the orientation of both cubes with respect to each other.

A block of unit voxels of size s_b contains s_b^3 unit voxels, and hence a similar number of ways exists to pair the central voxel with any of the voxels in the block. For each voxel pair the crossing probability of random line pieces in the central voxel with one voxel in the block was calculated. Fig. A6B displays these crossing probabilities for a block of size $s_b = 13$ μm (with in total $13^3 = 2197$ voxel pairs). The maximal distance between the central voxel and a corner voxel in the block was $d_{max} = 6\sqrt{3} = 10.39\ \mu m$. For overlapping voxels ($d_{v,v} = 0$) we had already (Eq. A6)

$$p^{cross}(d_{v,v} = 0) = 0.3133\ .$$

For non-overlapping voxels v and w ($d_{v,w} \geq 1$) the data points were fitted with a power law function

$$\log_{10} p^{cross}(d_{v,w} \geq 1) = a + b \times (x - c)^d \qquad \text{(A14)}$$

with best-fitting parameters a=32.75; b=-34.10; c=0.1966; and d=0.02383. Using the approximation $a^{\varepsilon} \cong 1 + \varepsilon \times lna$, we replaced the factor

$$(x - c)^d \cong 1 + d \times \ln(x - c) \qquad \text{(A15)}$$

resulting in

$$\log_{10} p^{cross}(d_{v,w} \geq 1) \cong$$
$$\cong -1.350 - 0.8126 \times \ln(d_{v,w} - 0.1966)$$

and

$$p^{cross}(d_{v,w} \geq 1) \cong$$
$$\cong 0.04467 \times (d_{v,w} - 0.1966)^{-1.8264}\ . \qquad \text{(A16)}$$

A4. CONDITIONAL CROSSING PROBABILITY

A4.1. CONDITIONAL CROSSING PROBABILITY OF RANDOM LINE PIECES IN A SINGLE CUBE OF ARBITRARY SIZE

Two random line pieces in a cube of size s_c will cross each other with a probability of (Eq. A12)

$$p^{cross} = 0.3133$$

and a mean crossing distance of (Eq. A11)

$$\overline{d^{cross}} = 0.334 \times s_c\ .$$

For the conditional crossing probability $p^{cross}(s_c|\delta)$ between random line pieces in a single cube, a constraint of δ μm is set on the maximal crossing distance between crossing line pieces.

6

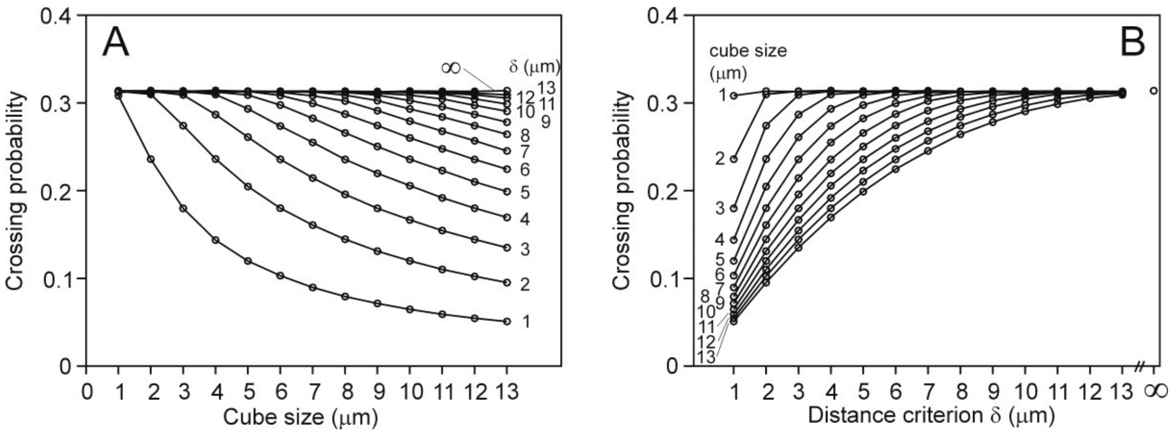

FIGURE A7 | Crossing probabilities of random line pieces in a cube. (**A**) Plotted versus the size of the cube (μm). Each curve is labeled with the value of the distance criterion δ (μm). (**B**) Plotted versus the distance criterion δ (μm). Each curve is labeled with the size of the cube (μm).

Thus only those pairs of crossing line pieces are selected that have a crossing distance that does not exceed the constraint of δ μm. This condition makes the conditional crossing probability dependent on the size s_c of the cube, as is shown graphically in Fig. A7. These outcomes are obtained by calculating crossing probabilities for a range of cube sizes of [1,13], for a range of criterion values δ of [1,13], and for the unconditional case (i.e., $\delta = \infty$). Each outcome is the mean value for a large number of random line piece pairs (n=1.000.000). Fig. A7 shows that the unconditional crossing probabilities ($\delta = \infty$) are independent of the size of the cube, as was already stated at Eq. (A12). The conditional crossing probabilities (finite δ), however, depend strongly on the cube size s_c. For instance, for $\delta = 1\,\mu m$, crossing line pieces are accepted only when their crossing distance is smaller than 1 μm. This strong requirement has the largest effect in large cubes, when on average the crossing distances vary over larger ranges (lowest curve in FigA7A).

A4.2. CONDITIONAL CROSSING PROBABILITY OF RANDOM LINE PIECES IN VOXELS AT A GIVEN DISTANCE

When a distance criterion δ is applied to the crossing probability of random line pieces in two different voxels v and w, the conditional crossing probability $p^{cross}(s_c, d_{v,w}|\delta)$ also becomes dependent on the distance $d_{v,w}$ between the two voxels (Fig. A8). These outcomes were obtained by taking random line pieces in all the ${s_c}^3$ unit voxels in a cube of size s_c and testing their crossing with random line pieces in the central voxel of the cube. Note that the size of the unit voxels is equal to 1 μm. Evidently, the conditional crossing probability for a voxel pair v,w at larger intervoxel distance $d_{v,w}$ than the criterion distance δ is zero,

$$p^{cross}(s_c, d_{v,w} \gg \delta|\delta) = 0\,. \qquad \text{(A17)}$$

Likewise, the conditional crossing probability of a voxel pair at smaller intervoxel distance than the criterion distance is equal to the unconditional crossing probability for that distance,

$$p^{cross}(s_c, d_{v,w} \ll \delta|\delta) = p^{cross}(d_{v,w})\,. \qquad \text{(A18)}$$

For this distance regime the curves in Fig. A8 are identical to the one in Fig. A6B. Voxel pairs at intervoxel distances around the criterion distance have lower conditional crossing probabilities,

$$p^{cross}(s_c, d_{v,w} \cong \delta|\delta) < p^{cross}(d_{v,w})\,. \qquad \text{(A19)}$$

For instance, for $\delta = 1\,\mu m$ only the voxels directly surrounding the central voxel contribute: there are 6 voxels at a distance of 1 μm, 12 voxels at a distance of $\sqrt{2}\,\mu m$, and 8 voxels at a distance of $\sqrt{3}\,\mu m$. Fig. A8 shows that for a given distance criterion δ, voxels at a maximal distance of $\delta + 1$ from the central voxel have a nonzero crossing probability. The sum of the crossing probabilities over all these voxel pairs in the block denotes the total expected number of crossings between random line pieces in the central voxel and in all the voxels in the local environment of the central voxel, as determined by the distance criterion δ. The continuous line in each panel indicates the cumulative sum with increasing voxel distance.

A5. OVERLAPPING DENSITY FIELDS

A5.1. EXPECTED NUMBER OF CROSSING LINE PIECES IN AN OVERLAP AREA OF TWO DENSITY FIELDS

Suppose we have two density fields D and A with densities ρ_D and ρ_A, respectively, with an area in space where they overlap. In this overlap area each voxel of size s will contain a mass $\rho_{vD} \times s^3$ from field D and a mass $\rho_{vA} \times s^3$ from field A. As discussed above, these masses can be related to the probabilities p_{vD}^{hit} and p_{vA}^{hit} that the voxel is intersected by lines of field D and field A, respectively.

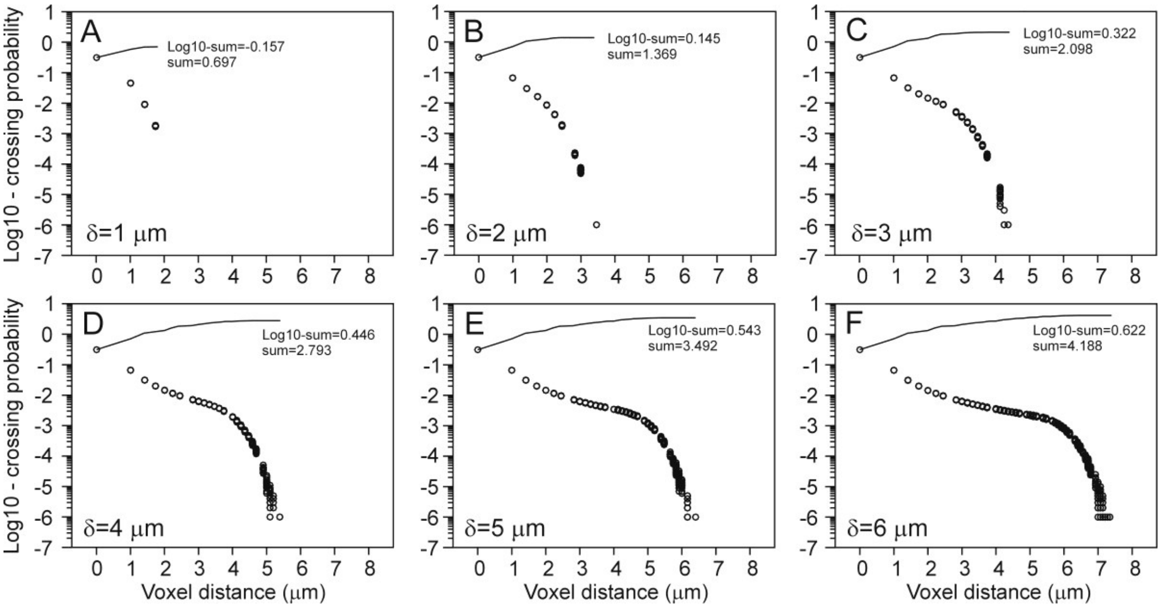

FIGURE A8 | Conditional crossing probabilities of random line pieces from a central voxel and one of the voxels in a block. Each data point indicates the crossing probability for one voxel pair, plotted versus the distance between both voxels. The total number of data points in a panel denotes the total number of voxel pairs with a positive crossing probability, i.e., of voxels sufficiently close to the central voxel, allowing crossing distances of line piece pairs within the distance criterion δ. Note that data points in the Figure may overlap. The sum of these crossing probabilities over all the voxels in the block denotes the total expected number of crossings between random line pieces in the central voxel and in all the voxels in the block. Each panel is labeled with the distance criterion value δ. The continuous line indicates the cumulative sum.

For any combination of line intersections of field *D* and field *A*, we now want to estimate the number of crossings line pieces over the given area in space. For a voxel pair *v* and *w* with an intervoxel distance $d_{v,w}$ the conditional weighted probability that a random line piece from field *D* in voxel *v* and a random line piece from field *A* in voxel *w* cross is given by

$$p_{v,w}^{cross}\left(s, d_{v,w} \middle| \delta\right) \times p_{vD}^{hit} \times p_{wA}^{hit} = \qquad \text{(A20)}$$

$$= p_{v,w}^{cross}\left(s, d_{v,w} \middle| \delta\right) \times \frac{\rho_{vD} \times s^2}{C} \times \frac{\rho_{wA} \times s^2}{C} =$$

$$= p_{v,w}^{cross}\left(s, d_{v,w} \middle| \delta\right) \times \rho_{vD} \times \rho_{wA} \times \frac{s^4}{C^2}.$$

The conditional weighted expected number of crossing line pieces of both fields in the overlap area can now be obtained by calculating the conditional weighted expected number of crossing line pieces that meet the distance criterion in all the voxel pairs in the overlap area:

$$E\{n_{D,A}^{cross} | \delta\} = \qquad \text{(A21)}$$

$$= \sum_v^{space} \sum_w^{space} p_{v,w}^{cross}\left(s, d_{v,w} \middle| \delta\right) \times \rho_{vD} \times \rho_{wA} \times \frac{s^4}{C^2} =$$

$$= \frac{s^4}{C^2} \times \sum_v^{space} \rho_{vD} \times \sum_w^{space} \rho_{wA} \times p_{v,w}^{cross}(s, d_{v,w} | \delta).$$

The summation in this expression runs over all voxel pairs in the given space. However, for each voxel of the field *D*, voxels of field *A* contribute positively to the summation only if the pairs of random line pieces do not cross beyond the criterion distance δ (Eqs. A17-A19). Thus the second summation over voxels *w* can be restricted to the local environment v_{env} of voxel *v*, resulting in the basic expression

$$E\{n_{D,A}^{cross} | \delta\} = \qquad \text{(A22)}$$

$$= \frac{s^4}{C^2} \times \sum_v^{space} \rho_{vD} \times \sum_w^{v_{env}} \rho_{wA} \times p_{v,w}^{cross}(s, d_{v,w} | \delta).$$

The local environment of voxel *v* with coordinates (i_v, j_v, k_v) can be defined as the set of all voxels *w* whose coordinates (i_w, j_w, k_w) meet the conditions

$$\begin{aligned} i_v - \delta &\le i_w \le i_v + \delta , \\ j_v - \delta &\le j_w \le j_v + \delta , \\ k_v - \delta &\le k_w \le k_v + \delta . \end{aligned} \qquad \text{(A23)}$$

A5.1.1. Overlap of individual neuron density fields

Equation (A22) can also be rewritten as the mean of the expected number of contacts in pairs of individual neurons. The densities ρ_{wA} and ρ_{vD} were obtained as the average over a population of soma-centered and aligned neurons:

$$\rho_{vD} = \frac{1}{n_D} \sum_i^{n_D} \rho_{vD_i} \text{ and } \rho_{wA} = \frac{1}{n_A} \sum_j^{n_A} \rho_{wA_j}.$$

Insertion in Eq. (A22) gives

$$E\{n_{D,A}^{cross}|\delta\} = \frac{s^4}{C^2} \times \sum_v^{space} \frac{1}{n_D} \times \sum_i^{n_D} \rho_{vD_i} \times$$

$$\times \sum_w^{v_{env}} \frac{1}{n_A} \sum_j^{n_A} \rho_{wA_j} \times p_{v,w}^{cross}(s, d_{v,w}|\delta)$$

and after exchange of summations

$$E\{n_{D,A}^{cross}|\delta\} = \frac{s^4}{C^2} \times \frac{1}{n_D} \times \frac{1}{n_A} \times \sum_i^{n_D} \sum_j^{n_A}$$

$$\sum_v^{space} \rho_{vD_i} \times \sum_w^{v_{env}} \rho_{wA_j} \times p_{v,w}^{cross}(s, d_{v,w}|\delta)$$

The last two summations result in the density overlap of the dendritic and axonal field of two individual neurons:

$$E\{n_{D,A}^{cross}|\delta\} = \frac{s^4}{C^2 \times n_D \times n_A} \times \sum_i^{n_D} \sum_j^{n_A} overlap(D_i, A_j) \quad \text{(A24)}$$

with

$$overlap(D_i, A_j) =$$

$$= \sum_v^{space} \rho_{vD_i} \times \sum_w^{v_{env}} \rho_{wA_j} \times p_{v,w}^{cross}(s, d_{v,w}|\delta) .$$

This result shows that the expected number of contacts obtained from the overlap of population mean density fields is equal to that obtained from the sum of the overlap of individual neuron density fields.

A5.2. APPROXIMATION – UNIFORMITY IN AXONAL DENSITIES IN THE LOCAL ENVIRONMENT OF DENDRITIC VOXELS

The distance criterion δ defines a maximal range around a dendritic voxel within which axonal voxels may contribute to synaptic contacts. In a highly irregular axonal density field (such as those of individual neurons), all the axonal voxels in the local environment must be included in the summation of (A22). However, a simplification of (A22) can be obtained if it can be assumed that the axon densities ρ_{wA} in voxels w in the local environment of a dendritic voxel v do not differ much from the axon density ρ_{vA} in the dendritic voxel v itself. In that case, Eq. (A22) can be approximated by

$$E\{n_{D,A}^{cross}|\delta\} \cong \quad \text{(A25)}$$

$$\cong \frac{s^4}{C^2} \times \sum_v^{space} \rho_{vD} \times \rho_{vA} \times \sum_w^{v_{env}} p_{v,w}^{cross}(s, d_{v,w}|\delta).$$

The second summation $\sum_w^{v_{env}} p_{v,w}^{cross}(s, d_{v,w}|\delta)$ runs over all the voxels w in the local environment of a given voxel v but does not depend on the position of voxel v. The second summation thus becomes a fixed number that is only dependent on the size of the voxels s and the distance criterion δ. It denotes the sum of unweighted conditional crossing probabilities of line pieces in a central voxel v and all the voxels w in the local environment of v that contribute to the sum, given the distance criterion δ. We will call this factor the *local environment crossing factor* $f^{env}(s, \delta)$:

$$f^{env}(s, \delta) = \sum_w^{v_{env}} p_{v,w}^{cross}(s, d_{v,w}| \delta) . \quad \text{(A26)}$$

Now we obtain from Eq. (A25)

$$E\{n_{D,A}^{cross}|\delta\} \cong \frac{s^4}{C^2} \times f^{env}(s, \delta) \times \sum_v^{space} \rho_{vd} \times \rho_{vA}. \quad \text{(A27)}$$

Thus the expected number of crossing line pieces between two density fields for a given value of the distance criterion δ is now approximated by the overlap sum of both fields $\sum_v^{space} \rho_{vd} \times \rho_{vA}$ (i.e., the sum of the density products per voxel over all voxels in the overlap area of space) multiplied with the coefficient $\frac{s^4}{C^2} \times f^{env}(s, \delta)$. If we denote the term $\sum_v^{space} \rho_{vd} \times \rho_{vA}$ in (A27) as the overlap sum I_{DA} of two overlapping density fields and the term $\frac{s^4}{C^2} \times f^{env}(s, \delta)$ as the overlap sum coefficient I_{coef} , we obtain from Eq. (A27)

$$E\{n_{D,A}^{cross}|\delta\} \cong I_{coef} \times I_{DA} \quad \text{(A28)}$$

with

$$I_{coef} = \frac{s^4}{C^2} \times f^{env}(s, \delta) \quad \text{(A29)}$$

and

$$I_{DA} = \sum_v^{space} \rho_{vD} \times \rho_{vA} . \quad \text{(A30)}$$

9

TABLE A1 | Local environment crossing factor $f^{env}(s, \delta)$ (sum of unweighted crossing probabilities of random line pieces in a central unit voxel and in all the unit voxels in a block of size s), with application of a distance criterion δ for the crossing distance between crossing line pieces. The bold values indicate the minimal block size needed for a given criterion value.

		Local environment crossing factor $f^{env}(s, \delta)$						
**s**	**# voxels**	**δ=∞**	**δ=1**	**δ=2**	**δ=3**	**δ=4**	**δ=5**	**δ=6**
1	1	0,3135	0,3087	0,3128	0,3131	0,3129	0,3129	0,314
3	27	1,2469	**0,6971**	1,1974	1,2476	1,2465	1,2477	1,2462
5	125	2,1121	0,6971	**1,3961**	1,9694	2,1083	2,1146	2,1113
7	343	2,9697	0,6971	1,3961	**2,0977**	2,6992	2,9445	2,9688
9	729	3,8262	0,6971	1,3961	2,0977	**2,7927**	3,4183	3,7384
11	1331	4,6800	0,6971	1,3961	2,0977	2,7927	**3,4920**	4,1272
13	2197	5,5348	0,6971	1,3961	2,0977	2,7927	3,4920	**4,1881**

A5.2.1. Local environment crossing factor $f^{env}(s,\delta)$ - sum of unweighted conditional crossing probabilities in the local environment of a given voxel v

For the local environment of a given voxel *v*, we will use a unit grid and take a cubic block of unit voxels of size s_b such that the maximal distance between the central voxel and a peripheral voxel is at least equal to $\delta+1$, as discussed following Eq. (A19). The size of the block should therefore be at least equal to $2\delta+1$. The unweighted sum of crossing probabilities (*local environment crossing factor* $f^{env}(s,\delta)$) is now obtained by summing the crossing probabilities for the central voxel paired with all the voxels in the block (A25). For results see Table A1. An illustration is given in Fig. A9, showing for a voxel block of size s_b=7 all the voxels and random lines in each of them. The central voxel is highlighted in red. The results are shown in Fig. A10A. For each value of the criterion δ, the curves show initially an increase with increasing values of the block size, but they become constant when the block size is equal to or larger than $2\delta+1$. For the unconditional case $\delta=\infty$ (when no distance criterion is imposed), the sum (i.e., the number of expected crossings) increases linearly with the size of the block for not too small block sizes.

A5.2.2. Local environment crossing factor $f^{env}(s,\delta)$ - local environment as a single cube of size equal to a block of voxels

In the previous section **A5.2.1**, the *local environment crossing factor* $f^{env}(s,\delta)$ (sum of unweighted crossing probabilities) was taken over the local environment v_{env} of a unit voxel *v*, in the form of a block of unit voxels of size $2\delta+1$ and centered at voxel *v*. When the axonal density field in the local environment of a dendritic voxel has a uniform density, then the axonal voxels in the local environment should be replaceable by a single axonal cube of the same size. Also the sum of crossing probabilities of random line pieces in the dendritic voxel and in all the local axonal voxels should be equal to the crossing probability of a random line piece in the dendritic voxel and a random line piece in the axonal cube. This consistency will be shown in the next two sections, the first one **(A5.2.3)** for the unconditional case (δ=∞) and the second one **(A5.2.4)** for the conditional case (δ=finite).

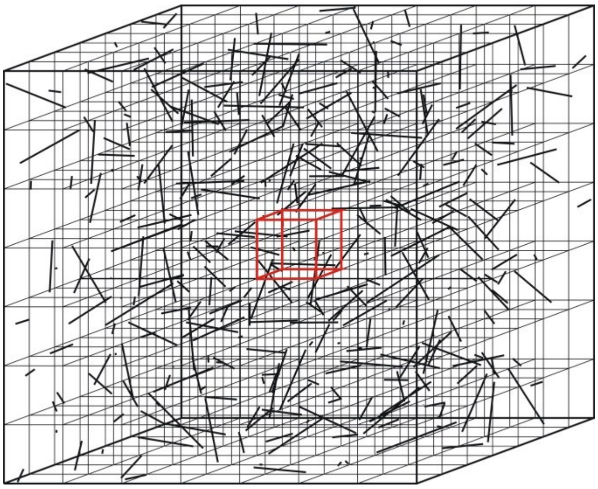

FIGURE A9 | A block of voxels of size s_b=7 with the central voxel highlighted in red. Each voxel contains a random line piece. Each of these random line pieces is tested for crossing with a (new) random line piece in the central voxel.

A5.2.3. Unconditional unweighted crossing probabilities of random line pieces in centered cubes of arbitrary size

To generalize the question, we will take two centered cubes of different sizes and determine whether random line pieces in both cubes are crossing. For example, Fig. A4B shows two centered cubes: a unit cube *v* and a larger cube *b*. A random line piece is drawn in both cubes. The probability of crossing was determined by taking a large number of line piece pairs (n=1000000) and counting the number of crossing line piece pairs. The crossing probabilities were obtained for different sizes of both cubes, i.e., for a cube *c1* with size *s1* and for a number of centered cubes *c2* with varying sizes *s2*. In the case of crossing line pieces, their crossing distance was also determined. The results are shown in the 3rd column of Table A2 and in Fig. A11.

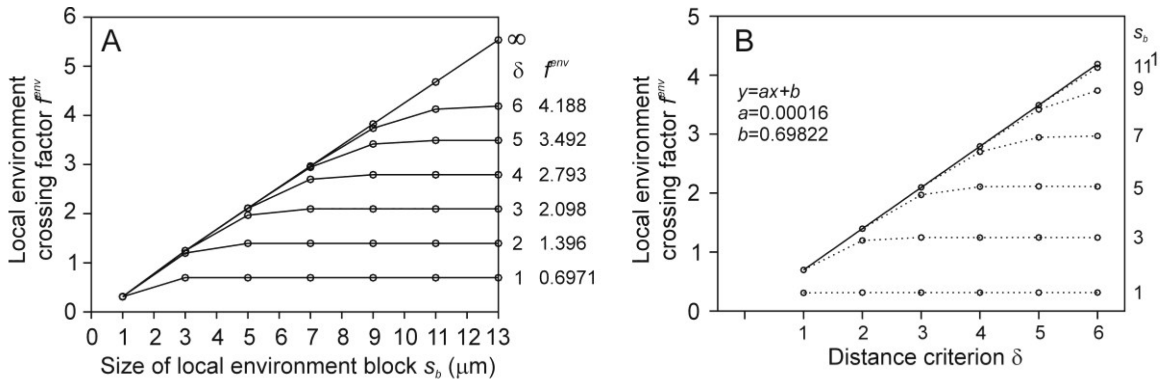

FIGURE A10 | Sum of unweighted conditional crossing probabilities of random line pieces in a central voxel and in each of the s^3 unit voxels in a centered block of size *s*. (**A**) Plot versus the size of the centered block s_b. The curves labeled with a positive value of δ indicate the summed unweighted conditional crossing probabilities. (**B**) Plot versus the distance criterion δ; the curves are labeled by the size of the block s_b. For each value of the distance criterion, the local environment factor has a maximum value, provided that the block size is of sufficient size. A linear fit through these maxima is drawn as a continuous line.

TABLE A2 | Unconditional crossing probabilities of random line pieces in centered cubes of different size (n=1000000). (1st column) Size cube 1; **(2nd column)** Size cube 2; **(3rd column)** Unconditional crossing probabilities; **(4th column)** Same values as in 3rd column but normalized for the density of line pieces; **(5th column)** Sum of crossing probabilities of random line pieces in a central voxel and in all the voxels in a block (cube2); **(6th column)** Difference between the values of the 4th and the 5th column.

size cube1	size cube2	pcross voxel block	pcross voxel block normalized	sum pcross block	difference
1	1	0,3124	0,3124	0,3135	-0,0011
1	3	0,1386	1,2472	1,2469	0,0003
1	5	0,0845	2,1119	2,1121	-0,0002
1	7	0,0603	2,9565	2,9697	-0,0132
1	9	0,0474	3,8418	3,8262	0,0157
1	11	0,0386	4,6702	4,6800	-0,0098
1	13	0,0330	5,5699	5,5348	0,0351

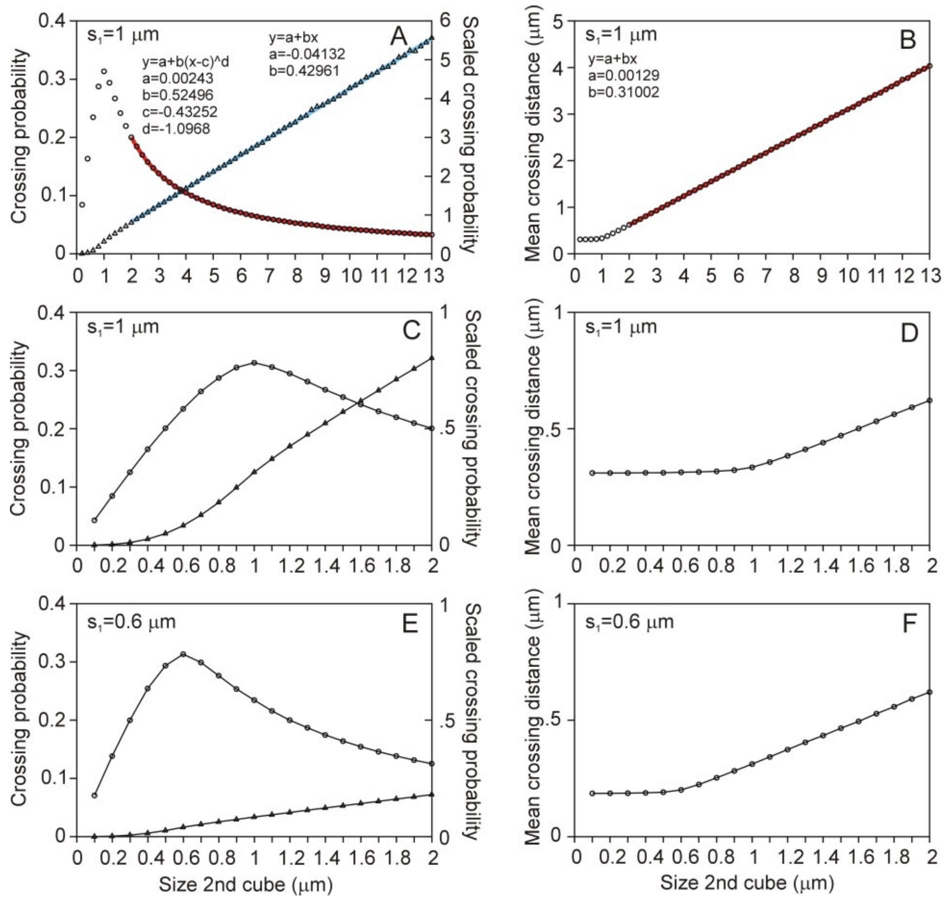

FIGURE A11 | Unconditional crossing probabilities of random line pieces in centered cubes of different size. (A) Octant data points show the crossing probability of random line pieces in cubes of size s_1=1 μm and of sizes s_2=0.2 - 13 μm, respectively. Triangle data points show the crossing probabilities, normalized for the size of s_2. The continuous lines through the data points for s_2>2 show the best fitting power function of the crossing probability data (red curve) and the best fitting linear function of the normalized data (blue curve). **(B)** Mean crossing distance between crossing line pieces. The continuous line through the data points for s_2>2 represents the best fitting linear function (red curve). **(C)** Octant data points show the crossing probability of random line pieces in cubes of size s_1=1 μm and of sizes s_2=0.1-2 μm, respectively; triangle data points show the crossing probabilities, normalized for the size of s_2. **(D)** Mean crossing distance between crossing line pieces. **(E)** and **(F)** are similar to **(C)** and **(D)** but with a size for cube 1 of s_1=0.6 μm. The continuous lines through the data points in panels **(C)**-**(F)** are linear interpolations between successive data points.

Figs. A11A, C, and E show that the crossing probability reaches the maximum value of 0.3133 when both cubes are of equal size (*s2=s1*) (Eq. A4):

$$p_{c1,c2}^{cross}(s_1 = s_2) = 0.3133 \ .$$

For $s_2 < s_1$ the crossing probability sharply declines, while for $s_2 > s_1$ the crossing probability gradually declines. For $s_2 \geq 2$, this dependency could well be fitted with a power law function

$$p_{c1,c2}^{cross}(s_1 = 1, s_2 \geq 2) = \qquad \text{(A31)}$$

$$= 0.00243 + 0.525 \times (s_2 + 0.433)^{-1.097} \ .$$

These results should now be compared with the summed crossing probabilities of line pieces from the central voxel and each of the voxels in the larger cube. These values were already calculated in the 3rd column of Table A1, for which no distance criterion was applied (δ=∞). These values are included in the 5th column of Table A2. For a proper comparison between the values in the 3rd and the 5th column of Table A2, a normalization is needed.

For the voxel-block calculations, single random line pieces are obtained in both centered cubes. A single random line in a cube represents a mass density equal to its length divided by the volume of the cube (Eq. A8). The mean length of a random line in a cube scales linearly with the size of the cube $\overline{L_c(s_c)} = C \times s_c$ (Eq. A4), while the volume of a cube scales as $V_c(s_c) = s_c^3$. The mass density in the cubes thus scales as $\rho_c(s) = \overline{L_c(s)}/V_c(s) = C/s_c^2$. For a valid comparison between the different sizes of the 2nd cube, we thus need to normalize for equal mass densities by multiplying the crossing probabilities with s^2. These normalized crossing probabilities, (Fig. A11A, triangle data points) appear to scale quite linearly with the size s_2 of cube 2 for s_2 values larger than about $2s_1$; for this range the triangle data points could well be fitted with a linear function

$$p_{c1,c2}^{cross}(s_1 = 1, s_2 \geq 2 | normalized) =$$

$$= -0.0413 + 0.4296 \times s_2 \ . \qquad \text{(A32)}$$

For single random lines, this means that the gradual decline of the crossing probability with increasing size of cube 2 is fully attributable to the decrease in mass density. The normalized values are also shown in the 4th column of Table A2. Now a comparison can be made between the voxel-block approach and the voxel-voxel sum approach. The differences between both, shown in the 6th column of Table A2, are very small, down to the precision of the calculations. Thus it can be concluded that both approaches give consistent results.

A5.2.4. Conditional weighted crossing probabilities of random line pieces in a central voxel and a centered cube

As we did for Eq. (A20), we may write the expected number of synaptic contacts $E_{vd}^{con}(\delta)$ of a dendritic line piece in a unit voxel *v* with axonal line pieces in the centered cube *c* for a given distance criterion δ, as the product

$$E_{vd}^{con}(\delta) = p_{vc}^{cross}(s_c|\delta) \times p_{vd}^{hit} \times p_{ca}^{hit} \qquad \text{(A33)}$$

with p_{vc}^{cross} the conditional crossing probability of random line pieces in voxel *v* and cube *c* of sufficient size s_c, p_{vd}^{hit} the probability that the central unit voxel *v* contains a dendritic line piece, and p_{ca}^{hit} the probablity that the centered cube *c* contains an axonal line piece. As in Eq. (A10), the hit probabilities p_{vd}^{hit} and p_{ca}^{hit} directly relate to the mass densities in the central unit voxel and the cube, respectively:

$$p_{vd}^{hit} = \frac{s_v^2 \times \rho_{vd}}{C} \quad \text{and}$$

$$p_{ca}^{hit} = \frac{mass_{ca}}{intersectionlength} = \frac{s_c^3 \times \rho_{ca}}{s_c \times C} = \frac{s_c^2 \times \rho_{ca}}{C} \ , \qquad \text{(A34)}$$

assuming a uniform axon density ρ_{ca} in the cube, which is thus equal to axon density in the central voxel *v* ($\rho_{ca} = \rho_{va}$). Insertion in Eq. (A33) now gives for the expected number of contacts

$$E_{vd}^{con}(\delta) \cong p_{vc}^{cross}(s_c|\delta) \times \frac{s_v^2 \times s_c^2}{C^2} \times \rho_{vd} \times \rho_{va} \ . \qquad \text{(A35)}$$

The conditional crossing probabilities $p_{vc}^{cross}(s_c|\delta)$ were obtained by simulating 1.000.000 pairs of random line pieces in the central voxel and in the centered cube, with results shown in Fig. A12 and listed in Table A3. For example, for a distance criterion of δ=4, and a cube size of s_c= 9 (=2δ+1), i.e., containing all the voxels that may contribute to connectivity with the central voxel, we obtained

$$p_{vc}^{cross}(s_c = 9 | \delta = 4, s_v = 1) = 0.0345 \qquad \text{(A36)}$$

and for the expected number of synaptic contacts with dendritic voxel *v*

$$E_{vd}^{con}(\delta = 4) \cong \frac{0.0345 \times 9^2}{0.66653^2} \times \rho_{vd} \times \rho_{va} =$$

$$= 6.290 \times \rho_{vd} \times \rho_{va} \ . \qquad \text{(A37)}$$

TABLE A3 | Conditional crossing probabilities between a random line piece in a central voxel and a random line piece in a centered cube

Distance criterion δ	Cube size s_c	Conditional crossing probability
1	3	0,0775
2	5	0,0556
3	7	0,0428
4	9	0,0345
5	11	0,0288
6	13	0,0247

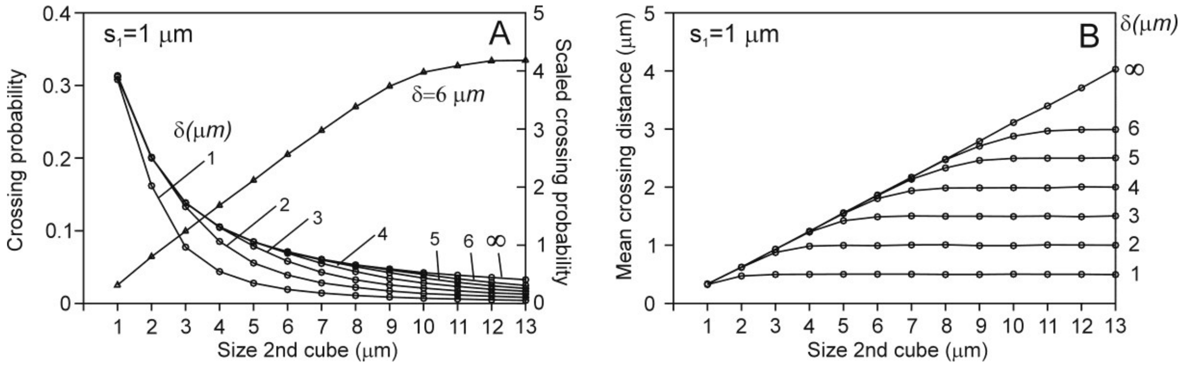

FIGURE A12 | (A) Octagon data points: unconditional (δ = ∞) and conditional (finite δ) crossing probabilities between random lines in a central voxel and in a centered block versus the size of the block (μm). The curves are labeled with the distance criterion δ. Triangle data points: conditional crossing probabilities scaled for the size of the 2nd cube, as shown for $\delta = 6\ \mu m$. This criterion value requires a block size of at least *s*=13 μm for collecting all possible contributions of crossing line pieces. **(B)** Conditional mean crossing distance between crossing line pieces in a central voxel and a centered block versus the size of the block. The curves are labeled with the distance criterion δ.

The coefficient in Eq. (A37) is equal to the overlap sum coefficient I_{coef} in Table A4 for $\delta = 4$; this also shows that the voxel-cube approach gives the same result as the sum of voxels in the block approach, under the assumption of a uniform axon density in the neighborhood of a dendritic voxel.

TABLE A4 | Overlap sum coefficient of two overlapping density fields

Distance criterion δ	Local environment factor $f^{env}(s = 1, \delta)$	Overlap sum coefficient I_{coef}
1	0.6971	1.569
2	1.396	3.142
3	2.098	4.723
4	2.793	6.287
5	3.492	7.860
6	4.188	9,427

A5.2.5. Overlap sum coefficient I_{coef} and closed expression of $E\{n_{D,A}^{cross}|\delta\}$

With the values for the local environment factor $f^{env}(s, \delta)$ (for *s*=1), the overlap sum coefficient I_{coef} can now be calculated explicitly using Eq. (A29) with the results given in Table A4. In Fig. A10B the local environment factor is plotted versus the distance criterion δ. For each value of δ, the local environment crossing factor has a maximum value, provided that the block size is of sufficient size. A linear fit through these maxima is drawn as a continuous line:

$$f^{env}(s = 1, \delta) \cong 0.00016 + 0.69822 \times |\delta| \qquad \text{(A38)}$$

with $|\delta|$ used here as a dimensionless variable. When an accuracy of at least 0.2 promille is sufficient, we may disregard the constant, so that the overlap sum coefficient I_{coef} can then be approximated by

$$I_{coef} \cong \frac{s^4}{C^2} \times 0.6982 \times |\delta| = 1.572 \times s^4 \times |\delta| \qquad \text{(A39)}$$

and the expected number of crossing line pieces in the overlap area is equal to

$$E\{n_{D,A}^{cross}|\delta\} \cong 1.572 \times |\delta| \times s^4 \times I_{DA}\ . \qquad \text{(A40)}$$

A5.3. CROSSING DISTANCES BETWEEN CROSSING LINE PIECE PAIRS IN CENTERED CUBES OF DIFFERENT SIZE

In the case of a crossing line piece pair, the crossing distance was also determined. The mean crossing distance between crossing line pieces for s_1=1 μm and different sizes s_2 of the 2nd cube appeared to scale linearly with s_2 for s_2>2 (Fig. A11B):

$$d_{mn}(s_2 = 1) = 0.334 \quad \text{(Eq. A11)}$$
$$d_{mn}(s_2 > 1) = 0.0013 + 0.310 \times s_2\ . \qquad \text{(A41)}$$

Both the crossing probability and the mean crossing distance for s_2=1 do not follow the scale behavior that is shown for s_2>1. To investigate this behavior in more detail, we performed similar calculations for sizes of cube s_2 between 0.1 and 2 (Fig. A11D) and for a smaller size of cube *s1* (0.6) (Fig. A11F).

As in Fig. A11A, also the data curves for the unnormalized crossing probabilities (i.e. of single line piece pairs) in Figs. A11C,E show the same maximum value of pcross=0.3133 when both cubes are of the same size. This is in agreement with the finding in Eq. (A12) that the crossing probability of line pieces within the same cube has a constant value of 0.3133, independent of the size of the cube. When the centered cubes are of different size, the line pieces

13

in a pair will on average be at a larger crossing distance from each other compared with line pieces within the same smaller cube. Thus, the crossing probability of random line pieces in centered cubes of different size will be smaller than the crossing probability of random line pieces in the same cube. The crossing distance curves (Figs A11B,D,F) also show a rather linear pattern when the 2nd cube becomes smaller than the 1st cube. The crossing distance stabilizes towards a value of about 0.31 when the 1st cube has size s_1=1µm, and towards a value of about 0.186 when the 1st cube has a size of s_1=0.6 µm. This can be understood by considering that a small 2nd cube is in the center of the 1st cube, and constrains one end of the orthogonal connection line between both line pieces to this small volume. The other end can be at any point within the larger cube. The length of the orthogonal connection line appeared to depend linearly on the size of the larger cube:

$$\lim_{s_2 \to 0} d_{mn}(s_1, s_2) = 0.31 \times s_1 \ . \qquad (A42)$$

Note that if one of the cubes becomes very small, also the crossing probability becomes very small.

Generalization

The crossing probability of two random lines within a cube appeared to be independent of the size of the cube, see Eq. (A12):

$$p^{cross}_{c1,c2}(s_1 = s_2) = 0.3133, \quad \text{and}$$
$$p^{cross}_{c1,c2}(as_1, as_2) = p^{cross}_{c1,c2}(s_1, s_2) \qquad (A43)$$

for any value of the constant a.

A6. INVARIANCE OF THE SCALE OF THE GRID FOR THE CALCULATION OF CROSSING PROBABILITIES

The scale of the grid, i.e., the size of the voxels, should be a free parameter in the discretization of space. However, the use of the distance criteron δ may set conditions on the choice of the grid size. The grid size is important for the spatial resolution of the density fields. Particularly in the case of density fields of individual neurons it sets a lower limit on the fine structure of individual branches. The procedure for calculating the crossing probabilities between random lines in different voxels is invariant to the scale of the grid, which can be proven as follows.

Given, a cube of arbitrary size s_c that is intersected by a random line from field A and a random line from field B. These random line pieces inside the cube will cross each other with a probability of (Eq. A12)

$$p^{cross} = 0.3133 \ ,$$

which is independent of the size s_c of the cube. These random line pieces have a mean length of $C \times s_c$ with $C = 0.66653$ (Eq. A4) which corresponds to a mass m_{cA} and m_{cB} with

$$m_{cA} = C \times s_c \ \text{ and } \ m_{cB} = C \times s_c \ .$$

Assuming uniformly distributed masses over the cube these two line pieces determine densities of (Eq. A8)

$$\rho_A = \frac{m_c}{s_c^3} = \frac{C}{s_c^2} \ \text{ and } \ \rho_B = \frac{C}{s_c^2} \text{, respectively.}$$

Crossing of random line pieces in all pairs of voxels in a block of unit voxels - When the cube is superposed by the unit grid with unit voxels ($s_v = 1$), random line pieces can be obtained in any voxel, given these field densities, with hit probabilities given by (Eq. A10)

$$p^{hit}_{vA} \cong \rho_A \times s_v^2 / C$$
and
$$p^{hit}_{vB} \cong \rho_B \times s_v^2 / C \ . \qquad (A44)$$

A random line piece in voxel v and in voxel w will cross each other with a probability of $p^{cross}_{v,w}(d_{v,w}|\delta)$, which is dependent on the distance $d_{v,w}$ between both voxels and the criterion value δ (Figs. A6 and A8). Summing these crossing probabilities of line pieces for all the voxel pairs in the cube gives an unweighted expected number of crossing line piece pairs

$$E_{unw}\{n^{cross}_{A,B}(cube|\delta)\} = \sum_v^{cube} \sum_w^{cube} p^{cross}_{v,w}(d_{v,w}|\delta). \qquad (A45)$$

The results of these unweighted expected number of crossings are shown in Table A5 for different cube sizes and criterion values.

TABLE A5 | Unweighted expected number of crossing line pieces in all voxel pairs in a cube

n=1000	δ				
cube size s_c	∞	1	2	3	4
1	0,313	0,319	0,293	0,304	0,315
2	4,969	3,761	5,076	4,945	5,003
3	25,433	14,467	21,973	25,243	25,401
4	80,235	36,974	60,685	73,167	78,856

Taking the hit probabilities into account gives the weighted expected number of crossing line piece pairs

$$E_w\{n^{cross}_{A,B}(cube|\delta)\} = \qquad (A46)$$
$$= \sum_v^{cube} \sum_w^{cube} p^{hit}_{vA} \times p^{hit}_{vB} \times p^{cross}_{v,w}(d_{v,w}|\delta).$$

Because the masses of both fields in the cube are distributed uniformly over all the voxels the voxel hit probabilities are equal for all the voxels. The quantity $p^{hit}_{vA} \times p^{hit}_{vB}$ can thus be put before the summation in Eq. (A46) with

TABLE A6 | Crossing probabilities of random line intersections of two fields *A* and *B* of a cube of size s_c=3 for all the 27^2 = 729 unit voxel pairs in the cube. (1st column) Distance criterion; **(2nd column)** Unweighted crossing probabilities of two random line intersections in the cube; **(3rd column)** Densities of field *A* and field *B* are taken so that the hit probability for the cube is equal to one; **(4th column)** Weighted crossing probabilities; **(5th column)** Sum of unweighted crossing probabilities of random line pieces in all voxel pairs in the cube; **(6th column)** Voxel hit probabilities for the given field densities; **(7th column)** Weighted sum of voxel-voxel crossing probabilities.

s_{cube}=3 ncnt=100000						
δ	p^{cross} cube	p^{hit} cube	p^{cross} cube hit	sum p^{cross} vox-vox	p^{hit} voxel	sum p^{cross} vox-vox hit
∞	0,3138	1	0,3138	25,3566	0,1111	0,3130
1	0,1806	1	0,1806	14,5558	0,1111	0,1797
2	0,2742	1	0,2742	22,2021	0,1111	0,2741
3	0,3099	1	0,3099	25,0197	0,1111	0,3089
4	0,3132	1	0,3132	25,3370	0,1111	0,3128

$$E_w\{n_{A,B}^{cross}(cube|\delta)\} = p_{vA}^{hit} \times p_{vB}^{hit} \times$$

$$\times \sum_v^{cube} \sum_w^{cube} p_{v,w}^{cross}(d_{v,w}|\delta) =$$

$$= p_{vA}^{hit} \times p_{vB}^{hit} \times E_{unw}\{n_{A,B}^{cross}(cube|\delta)\} \quad (A47)$$

with the voxel hit probabilities p_{vA}^{hit} and p_{vB}^{hit} given by Eq. (A44). For instance, for a cube of size s_c=3, the field densities become $\rho_A = \rho_B = C/s_c^2 = 0.074$, and the hit probabilities $p_{vA}^{hit} = p_{vB}^{hit} = 0.1111$, so we obtain

$$E_w\{n_{A,B}^{cross}(cube|\delta)\} = 0.01234 \times$$

$$\times E_{unw}\{n_{A,B}^{cross}(cube|\delta)\} =$$

$$= 0.01234 \times 25.3566 = 0.3129 \quad (A48)$$

(see Table A6). This outcome is equal to the expected value of $p^{cross} = 0.3133$ of the two random line pieces in the whole cube which proves that the procedure for calculating the crossing probabilities between line intersections is invariant for the scale of the spatial grid. This conclusion holds also for other values of δ as is shown in Table A6.

A7. ESTIMATING THE CONNECTION PROBABILITY AND THE EXPECTED NUMBER OF CONTACTS PER CONNECTED NEURON PAIR FROM THE EXPECTED NUMBER OF SYNAPTIC CONNECTIONS

Let area *A* be the overlap area of an axonal and a dendritic density field, containing an expected number of synaptic connections $E\{n_A^{syn}\}$. The connection probability p_A^{con} that the axonal and dendritic neuron are connected (i.e., have at least one synaptic contact) can be estimated theoretically from the expected number of synaptic connections $E\{n_A^{syn}\}$ in the following way. Partition the overlap area *A* into a number of *C* small (for instance cubic) compartments. Distribute in an arbitrary way (say uniformly) the expected number of synapses $E\{n_A^{syn}\}$ over these compartments, so that

$$E\{n_c^{syn}\} = \frac{1}{C} E\{n_A^{syn}\} . \quad (A49)$$

Make the number of compartments so large that the expected number of synapses per compartment $E\{n_c^{syn}\}$ becomes much smaller than one. Then this value per compartment can be interpreted as the probability p_c^{syn} of finding a synapse in that compartment. The probability p_c^{nosyn} of not finding a synapse in that compartment is then given by $p_c^{nosyn} = 1 - p_c^{syn}$. The product of the no-synapse probabilities of all compartments in area *A*, assuming independency, then yields the probability of no-synapse in the overlap space, $p_A^{nosyn} = \prod_i^C (1 - p_{c_i}^{syn})$. The connection probability p_A^{con}, i.e., the probability of at least one contact in the overlap space, is then given by $p_A^{con} = 1 - p_A^{nosyn}$.

The expected number of contacts per connected neuron pair $E\{M\}$ is given by the ratio of the expected number of contacts in the overlap area divided by the connection probability,

$$E\{M\} = E\{n_A^{syn}\}/p_A^{con} . \quad (A50)$$

This relation can be derived as follows: Let $n(i)$ denotes the number of neuron pairs connected with *i* contacts. The total number of pairs is then given by $n = \sum_{i=0}^{i_{max}} n(i)$, while $p(i) = \frac{n(i)}{n}$ denotes the probability that a given neuron pair has *i* contacts. The expected number of contacts in an arbitrary neuron pair is given by $E\{i\} = \frac{1}{n}\sum_{i=0}^{i_{max}} i \times n(i) = \sum_{i=0}^{i_{max}} i \times p(i)$. The connection probability is given by $p^{con} = \frac{1}{n}\sum_{i=1}^{i_{max}} n(i) = \frac{n-n(0)}{n}$. The expected number of contacts per connected neuron pair becomes $E\{M\} = \sum_{i=0}^{i_{max}} i \times n(i) / \sum_{i=1}^{i_{max}} p(i) = \sum_{i=0}^{i_{max}} i \times n(i)/n - n(0) = = \frac{n \times E\{i\}}{n-n(0)} = \frac{E\{i\}}{p^{con}}$, which proves Eq. (A50).

Fig. A13 shows how the connection probability and the expected number of contacts per connection depend on the expected number of contacts. The procedure for estimating the connection probability is independent of the number of compartments or the way the quantity $E\{n_c\}$ is distributed over the compartments, as long as the values per compartment remain much smaller than one, and independency can be assumed between the compartments. However, as is shown in the section **ESTIMATION OF THE CONNECTION PROBABILITY FROM THE EXPECTED NUMBER OF CONTACTS** of the paper, synapse locations are not independently distributed in space, and the empirical relationships between number of contacts on the one hand, and connection probability and number of contacts per connection on the other hand, are different from the curves in Fig. A13 (see also Figs. 6 and 8 in the paper).

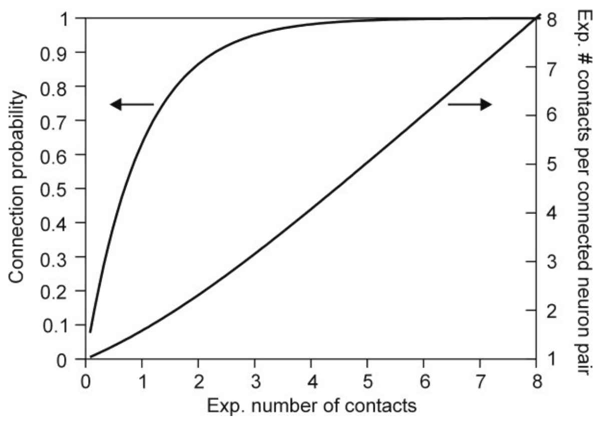

FIGURE A13 | Connection probability and expected number of contacts per connected neuron pair derived from the Expected number of contacts.
